# A Multi-Layer Techno-Economic-Environmental Energy Management Optimization in Cooperative Multi-Microgrids with Demand Response Program and Uncertainties Consideration

**DOI:** 10.1038/s41598-024-72706-3

**Published:** 2024-10-08

**Authors:** Nehmedo Alamir, Salah Kamel, Tamer F. Megahed, Maiya Hori, Sobhy M. Abdelkader

**Affiliations:** 1 Electrical Power Engineering, Egypt-Japan University of Science and Technology, 21934 New Borg El-Arab City, Egypt; 2 Department of Electrical Engineering, Faculty of Engineering, Aswan University, 81542 Aswan, Egypt; 3 Department of Electrical Engineering, Faculty of Engineering, Mansura University, 35516 Mansoura, Egypt; 4 Tottori University of Environmental Studies, 1-1-1 Wakabadai-kita, Tottori, 689-1111 Japan

**Keywords:** Enhanced equilibrium optimizer, Multi-Microgrid, Multi-layer multi-objective optimization, ${\epsilon}$ε-lexicography approach, 2m + 1 point estimation method, Propalistic, Electrical and electronic engineering, Renewable energy

## Abstract

This paper presents a multi-layer, multi-objective (MLMO) optimization model for techno-economic-environmental energy management in cooperative multi-Microgrids (MMGs) that incorporates a Demand Response Program (DRP). The proposed MLMO approach simultaneously optimizes operating costs, MMG operator benefits, environmental emissions, and MMG dependency. This paper proposed a new hybrid ε-lexicography–weighted-sum that eliminates the need to normalize or scalarize objectives. The first layer of the model schedules MMG resources with DRP to minimize operating costs (local generation and power transactions with the utility grid) and maximize MMG profit. The second layer achieves the environmental operation of the MMG, while the third layer maximizes MMG reliability. This paper also proposed a new application of a recently developed enhanced equilibrium optimizer (EEO) for solving the three-layer EM problem. In addition, the uncertainties of solar power generation, wind power generation, load demand, and energy prices are considered based on the probabilistic 2m + 1 Point estimation method (PEM) approach. Three case studies are presented to verify the proposed MLMO approach on an MMG test system. In Case I, a deterministic EM is solved to simulate the MMG as a single layer to minimize costs and maximize benefits through DRP, while Case II solves the MLMO optimization problem. Simulation results show that the proposed MLMO technique reduces environmental emissions by 2.45% and 3.5% in its optimization layer and at the final layer, respectively. The independence index is also enhanced by 2.49% and 4.8% in its layer only and as a total increase, respectively. Case III is for the probabilistic EM simulation; due to the uncertain variables effect, the mean value in this case is increased by about 2.6% over Case I.

## Introduction

### Motivation

Nowadays, most electrical energy is produced through conventional power plants that operate on a large scale. This method of power generation is associated with significant electrical losses, as well as high levels of environmental emissions resulting from the combustion of fossil fuels, and a lack of dependability^1,2^. Integrating low-carbon renewable energy sources effectively address environmental and energy issues and enhances the system’s operational flexibility^3,4^. Microgrid (MG), which is considered the basic building block of the Smart Grid (SG), enables the effective control and utilization of RES and the effective control of responsive loads^5–7^. However, due to the challenging energy management (EM) process of MG resources^8^ and the uncertainties existed in renewable energy sources (RES), the bulk integration of these resources in a Single MG (SMG) is infeasible^9^. The networked MG or multi-Microgrid (MMG) is a cluster of connected MGs that help integrate many DERs via local integration^10,11^. Authors in^12^ proposed a modified version of Slime Mould Algorithm (SMA) and demand response (DR) technique to minimize the operating cost and for the peak load reduction or load curve shaping. MMG mitigates the issues related to the bulk integration of RES, also, due to the recent development of the communication infrastructure of SG^13^, the focus has been shifted from SMG to MMG^14^.

The MMG resources should be managed and coordinated in a manner to achieve a techno, economic, and stable operation. In addition, energy management system (EMS) helps in maximizing the utilization of power generated from renewable and clean energy resources. Therefore, an effective EMS is necessary to achieve these multiple MMG objectives. The EMS in MMG can be categorized into two main groups: Competitive or non-cooperative EMS, and Collaborative or cooperative EMS. In the first category, each SMG strives to achieve its own objectives, while in the latter, SMGs collaborate to attain some common objectives such as enhancing the MMG reliability, security, and resilience on the utility grid (UG)^15–17^.

Recently, the integration of ancillary services in the EMS of MMGs has received great attention due to the advancement of the responsive metering devises in SG, which helps enhancing the techno-economic operation^18^. Demand Side management, including demand response, has emerged as an ancillary services tool that can be integrated in the EMS of MMG which manages the end-customers’ consumption based on different pricing techniques. DR is categorized into: Price based DR (PBDR), where the prices are fluctuated with time, and incentive Based DR (IBDR), where the customers are incentivized for their consumption reduction^19^.

In Power systems, different variables have an uncertain nature. It is essential to consider these uncertainties in molding for better decisions. The common strategies for uncertainty consideration include probabilistic and possibilistic approaches; probabilistic such as Monte Carlo simulation (MCS), scenario-based analysis (SBA), and Point estimate method (PEM), and the possibilistic fuzzy membership function is used for uncertain parameters, and it is dealt by fuzzy arithmetic.

### Related work

In the last decade, several researchers have concentrated on studying the EM of MG. The optimal EM of SMG is proposed in^20^ for cost and energy not supplied (ENS) minimization and battery usage maximization. The uncertainties in energy cost, load demand and wind generation were not considered. In ref^21^, a stochastic 2 m + 1 PEM to minimize the operating cost using Particle swarm optimization(PSO) of renewable-based SMG; however, the reliability and the environmental effect were not considered. The EMS solves a multi-objective problem based on the weighted-sum technique for operating cost minimization and the MG operator (MGO) benefit maximization in^22^ based on Honey badger Algorithm, the EM problem is solved without the consideration of the uncertain nature of MG resources. In^23^ different optimization techniques such PSO, GA, evolve class topper optimization, and red deer algorithm (RDA) to optimize the cost in a two-level techno-economic analysis for the integrated energy systems. In^24^, using Pelican Optimization Algorithm (POA) and weighted-sum technique a deterministic hybrid DRP technique is proposed for cost minimization, benefits maximization, and peak load reduction; however, the probabilistic EM was not involved. The author used an Advanced Interactive Multidimensional Modelling System (AIMMS) in^25^ to solve deterministic multi-objective optimization based on weighted–sum technique and the consideration of DR to minimize the cost and enhance the MGO profit. In^26^ a Spotted hyena optimizer (SHO) is used for optimal for cost minimization and capacitor allocation in MG. All these researches are limited to SMG only.

The US Department of Energy (DOE) stated that SMGs will be the essential building blocks of the future electricity system to enhance reliability and achieve decarbonization by 2035^27^. This increase in SMG numbers led to an increase in the interaction between SMGs, so the MMG concept emerged.

The planning and operation of either non-cooperative (NCO)^28–32^ or cooperative (CO)^33,34^ MMG is a hot research topic that has been discussed in many research works^16^. In^13^, an overview of the benefits and difficulties of MMGs is provided. A non-cooperative EM in MMG using the Nash Equilibrium concept and an iterative method is proposed in^28^ to manage the power-sharing for each SMG; however the demand response and the reliability of MMG was not considered. Author in^29^ use a two-stage analytical target cascading (ATC) theory based for minimizing MMG cost and the cost of power purchased from the connected distribution network (DN) independently; the DRP and uncertainties were not considered in the EMS. A non-cooperative MMG based on Multi-agent system (MAS) was proposed in^30^to increase the profit of MMGs and to enhance the reliability. The uncertainties based on scenario generation and reduction method using the Latin hypercube sampling (LHS) technique were implemented. However, this paper did not consider the DRP integration and the MMG dependability from the utility grid. Ref^31^. uses MLIP with a lexicography approach and Compromise Programming (CP) to solve a two-stage multi-objective optimization (MOO) problem in non-cooperative MMG. The profit of the connected distribution network is maximized in the first stage by considering the ENS minimization, while in the operating cost is minimized. Scenario generation analysis technique based on discretization of the Probability density function (PDF) was implemented for the stochastic EM. The environmental effect and the DRP were not discussed. In^32^ a deterministic dynamic EMS based on an improved sparrow search algorithm (ISSA) is proposed for non-cooperative MMG. The proposed model is to achieve an economic benefit for both MMG and the Active distribution network.

Ref.^33^ uses the alternating direction method of multipliers (ADMM) in the EMS in cooperative MMG to reduce the operating cost and emissions by integrating the IBDR program; the uncertainty of energy prices and load-supply mismatch were considered; however, the dependability from the utility grid was not considered. Using GAMS software, a multi-energy MMG a MOO problem based on the epsilon-constraint technique is formulated as a deterministic mixed-integer non-linear programming (MINLP) problem and solved in^34^. The objectives of the EMS are to minimize the operating cost and the amount of portable water extracted from water wells. However, dependability and the uncertainties were not considered. A cooperative two-stage EMS for MMG is proposed in^35^. The first stage is to minimize the operating cost in day-ahead EM, while the second stage is to consider the fluctuation of RES in real-time while minimizing the operating cost. The problem is formulated as MILP, and a discretization SG-SR technique for the stochastic EM were implemented; the DRP is considered to minimize the peak load; however, the emissions and the MMG dependability were not considered. In^36^, a cooperative MMG is proposed, and the EM problem is solved based on the weighted-sum technique and Snake Optimizer as a deterministic formulation. The objective is to maximize the benefit from DRP and minimize the operating cost; the independence index is evaluated based on the integration of DRP; however, the environmental effect was not considered. Authors in^37^ proposed a distributed EMS in cooperative MMG based on open-source MILP in order to minimize the operating cost; however, the environmental effect, DR, and the IPI were not considered. In^38^, the EMS, in cooperation with MMG based on multi-agent, is proposed to ensure lower data usage, and self-sufficiency maintain higher reliability. A cooperative EMS in MMG with an Energy storage system was discussed in^39^. A two-stage optimization problem is solved using a combined lexicography-fuzzy approach. The first stage minimizes the operating cost, and imported as constraint to the second stage. The second stage is to minimize pollution and maximize the Average Reserve Index (IRI) by maximizing the energy stored in the energy storage system. The sizing of DER and ESS in cooperative MMG is considered by solving a multi-objective problem based on PSO^40^ The objectives are to minimize the Levelized cost of energy and power losses cost. The DR, IPI, and uncertainty consideration were not integrated into EMS. A comparative analysis of the related work is illustrated in Table 1.

In solving the optimization problem, Deterministic algorithms can effectively solve a large number of problems; however some of these methods are very slow and requires many evaluation functions for the system to converge. Also, deterministic algorithms often require derivative information and tend to get stuck in locally optimal solutions. Consequently, they struggle when confronted with highly constrained, complex problems featuring multiple peaks^41,42^. The non-linear and non-convex programming has disadvantages of being complex in design and has convergence problems such as Newton based techniques; in interior point method.

Metaheuristic techniques have emerged as a promising solution to tackle these limitations. Metaheuristic methods employ various operators iteratively to explore and exploit the search space based on optimization objectives. These algorithms strike a balance between exploration and exploitation, allowing them to efficiently tackle a wide range of optimization problems. This was the main inspiration for us to select the metaheuristic techniques to be implemented to solve the EM problem. In addition, No-Free-Lunch (NFL) theorem^43^ stated that no metaheuristic optimization algorithms can solve every optimization problem. These inspire the researcher to develop new optimization algorithms. Also, this is the main motivation for us to employ the newly-developed algorithm, enhanced equilibrium optimizer (EEO), for solving the scheduling problem in MMG.

**Table 1:** Comparative summary of this study and previous works.

Ref	MG type	Formulation	Objectives	Uncertainty	DR	POL	IPI
^20^	SMG	• Multi-objective	• Cost minimization • ENS minimization • Battery life maximization	**✘**	**✓**	**✘**	**✘**
^21^	SMG	• Single objective • Metaheuristics (PSO)	• Cost minimization • Emissions minimization	**✓** (2 m + 1)	**✘**	**✘**	**✘**
^22^	SMG	• Multi-objective • Weighted-sum technique • Metaheuristics (HBA)	• Cost minimization • Profit maximization	**✘**	**✓**	**✘**	**✘**
^24^	SMG	• Multi-objective • Weighted-sum technique • Metaheuristics (POA)	• Cost minimization • profit maximization • peak load reduction	**✘**	**✓**	**✘**	**✘**
^25^	SMG	• Multi-objective	• Cost minimization • profit maximization	**✘**	**✓**	**✓**	**✘**
^26^	SMG	• Metaheuristics (SHO) • Single objective	• Cost Minimization	**✘**	**✘**	**✘**	**✘**
^29^	NCO-MMG	• Multi-objective • Two level optimization • ATC	• Cost of minimization • Cost of power purchased minimization • Cost of battery operation	**✘**	**✘**	**✘**	**✘**
^30^	NCO- MMG	• Multi-objective • Bi-level • MAS	• MMG’s Cost minimization • DN’s Cost minimization	**✓** (LHS)	**✘**	**✘**	**✘**
^31^	NCO- MMG	• Multi-objective • Two level • lexicography approach-CP • MLIP	• Cost minimization • Reliability enhancement	**✓** (SBA)	**✘**	**✘**	**✘**
^32^	NCO- MMG	• Multi-objective • Two-stage • Metaheuristic (ISSA)	• Cost Minimization • Profit of DN maximization	**✘**	**✘**	**✘**	**✓**
^33^	CO-MMG	• Multi-objective • ADDM	• SMG cost minimization • Emissions minimization	**✓**	**✘**	**✓**	**✘**
^34^	CO-MMG	• Multi-objective • epsilon-constraint • GAMS	• Cost minimization • Emissions minimization	**✘**	**✓**	**✓**	**✓**
^35^	CO-MMG	• Single objective • Two-stage	• Cost minimization	**✓** (SBA)	**✓**	**✘**	**✘**
^36^	CO-MMG	• Multi-objective • Weighted-sum • Metaheuristics SO	• Cost minimization • benefit maximization	**✘**	**✓**	**✘**	**✓**
^37^	CO-MMG	• Single • MILP	• Cost minimization	**✘**	**✘**	**✘**	**✘**
^39^	CO-MMG	• Two-stage • Multi-objective • lexicography approach -Fuzzy	• Cost minimization • Emissions minimization • IRI maximization	**✓** (SBA)	**✓**	**✓**	**✘**
^40^	CO MMG	• Multi-objective • Metaheuristics (PSO)	• Levelized cost minimization • power losses minimization	**✘**	**✓**	**✓**	**✓**
Proposed	CO MMG	• Metaheuristic EEO • Proposed Hybrid ε-lexicography –weighted-sum	• Cost minimization • Emissions minimization • Benefit maximization • Independence maximization	**✓**	**✓**	**✓**	**✓**

### Contribution

The existing literature on EMS in MMG primarily focuses on minimizing costs while giving less attention to other objectives such as minimizing power loss, ENS, emissions, and maximizing IPI. However, optimizing these objectives using the Pareto principle often leads to increased costs. Therefore, this paper aims to introduce a collaborative multi-layer, multi-objective model for the EMS in grid-connected MMG. The proposed model minimizes operating costs, including generation and energy transaction costs with the UG, and maximizes MGO profits. It also incorporates an IBDR program to enhance the MMG’s reliability while minimizing greenhouse gas emissions. The paper presents the first MLMO model that considers techno-economic-environmental factors in MMG using a hybrid lexicography-weighted-sum technique and integrates DR to maximize MGO benefit simultaneously. In addition the generation, demand, and energy prices uncertainties are considered based on PEM approache. The main contributions of this study are summarized as:

- Proposing a multi-layer, multi-objective model for the EMS in MMG that simultaneously considers the minimization of operating costs, maximization of MGO profit, reduction of greenhouse gas emissions, and enhancement of UG dependency.
- Developing a three-layer model that optimizes the operation of MMG resources to achieve specific objectives. The first layer focuses on minimizing the economic operation of MMG, the second layer addresses environmental concerns, and the third layer enhances independence. This approach eliminates the need for normalization or scalarization of objective functions.
- Introducing a new application of the Enhanced Equilibrium Optimizer (EEO) to solve the EM problem in the multi-layer, multi-objective EMS.
- Solving a stochastic EM problem at the uncertainties of renewable generation output, load demand, and energy prices based on PEM.

### Paper organization

The structure of the remaining sections of this paper is as follows: in Section "MMG Model description", MMG structure and modeling are discussed. Section Techno-economic environmental multi-layer multi-objective energy management presents the proposed MLMO energy management system for the MMG. The probabilistic EM formulation is discussed in Section "Uncertainty Modeling" . The fundamental of the EEO algorithm is presented in Section ‎"Solution method". The simulation results are presented in Section MMG Model description, and finally, the paper is concluded in section ‎"Conclusion".

## MMG Model description

**Fig. 1:**
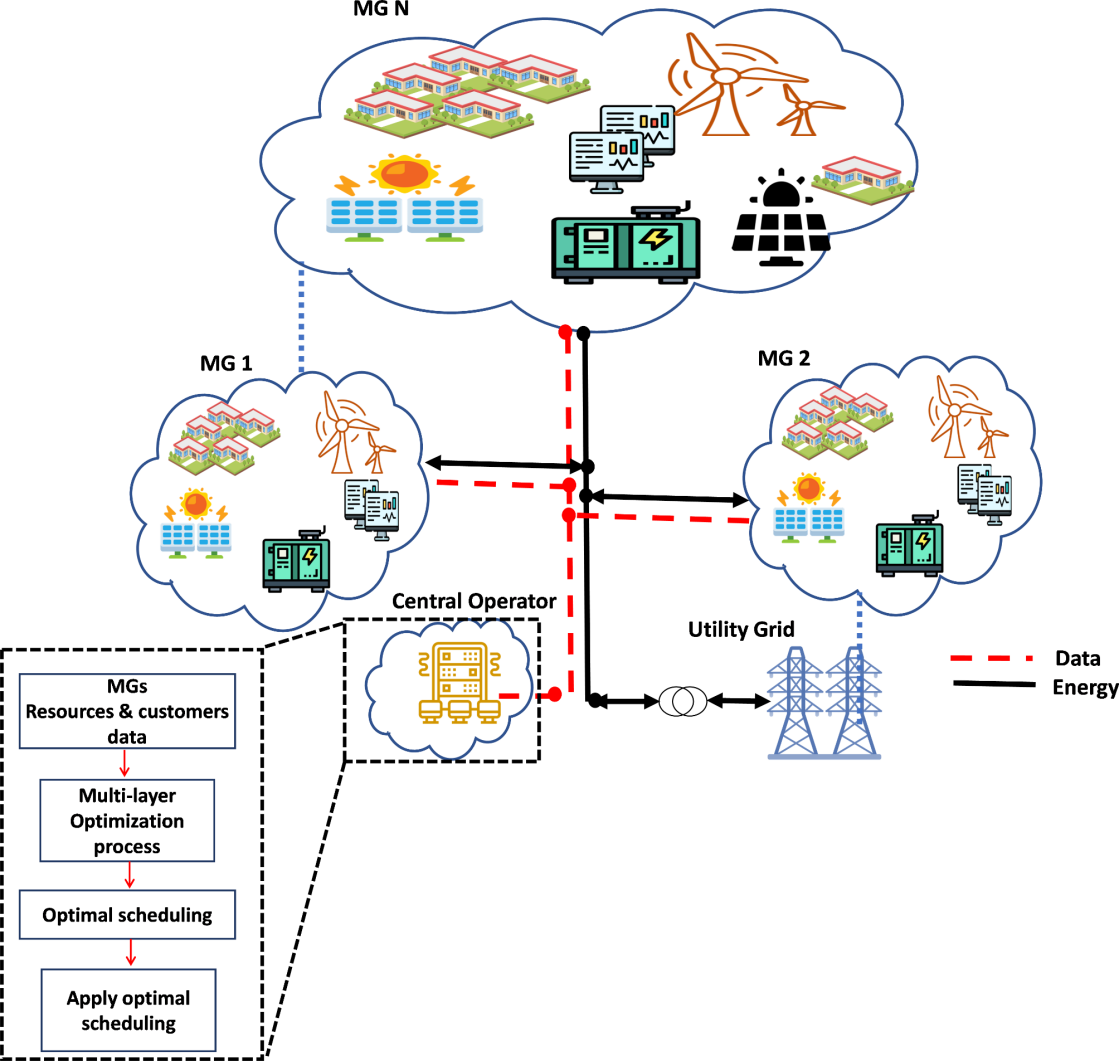
MMG architecture.

This paper proposes an MLMO model for EMS in a cooperative MMG. In the proposed model, SMGs interact to form one collection of MMG. In this collaboration, SMGs share their resources to minimize the operating cost. Also, the energy can be transacted with the utility grid. The EM is the Central Operator (CO) responsibility. Figure 1 illustrates the proposed architecture of the MMG. In this architecture, MGs share their resources and customer data to the CO before the CO determines the optimal operation of all MMG resources, a deataled EM problem formulation will be discussed in Sect. Related work. An IBDR is integrated into MMG’s EMS; so the customers can participate through reducing their consumption via DRP. The objectives of the EMS cost reduction, CO benefit increase, emission reduction, and independence of MMG increase; so for dealing with all nonhomogeneous objectives multi-layer multi-objective optimization model is proposed, and the solution procedure is described in Section ‎"Techno-economic environmental multi-layer multi-objective energy management".

### Grid connection model

The MMG is connected to the UG and the power can be transacted between them. The cost of power transaction with the grid at any time interval $\text{t}$ can be expressed as^25^:

1 $$\:cos{t}_{t}^{UG}\left({P}_{U{G}_{t}}\:\right)=\:\:\:\left\{\left.\begin{array}{c}{\gamma\:}_{t}\times\:\left|{P}_{U{G}_{t}}\right|\:\:\:\:\:\:\:\:\:\:\:\:\:\:\:\:\:\:\:\:\:\:\:\:\:\:From\:UG\:to\:MMG\\\:0\:\:\:\:\:\:\:\:\:\:\:\:\:\:\:\:\:\:\:\:\:\:\:\:\:\:\:\:\:\:\:\:\:\:\:\:no\:transaction\\\:{-\gamma\:}_{t}\times\:\left|{P}_{U{G}_{t}}\right|\:\:\:\:\:\:\:\:\:\:\:\:\:\:\:\:\:\:\:\:\:\:\:from\:MMG\:to\:UG\end{array}\right\}\right.$$

Where ${\text{P}}_{{\text{U}\text{G}}_{\text{t}}}$ is the amount of power transacted with UG, ${{\upgamma\:}}_{\text{t}}$ is the Locational Marginal Prices (LMP’s).

### Conventional generator models

A diesel generator (DG) is one of the dispatchable generation sources that the CO can adjust its output flexibly. The cost of power generation for generator $\:i$ ($\:cos{t}_{i}^{DG}\left({P}_{{i}_{t}}\:\right))$ can be mathematicaly expressed as^44^:

2 $$\:cos{t}_{i}^{DG}\left({P}_{{i}_{t}}\:\right)={a}_{i}{{p}^{2}}_{{i}_{t}}+{b}_{i}{P}_{{i}_{t}}$$

Where $${P}_{{i}_{t}}\:$$is the power output from DG $$\:i$$ at any time interval $$\:t$$$$\:,{\text{a}}_{\text{i}}$$ and $$\:{b}_{i}\:$$represents its fuel cost coefficients.

### Incentive demand response program model

The customers in MMG can participate actively through the DRP; they get signals from the CO to start to reduce or curtail their consumption in response to an incentive payment they will get.

The cost function of $\:{j}^{th}$ customer can be mathematically expressed as:

3 $$\:Cos{{t}_{j}}^{cust}\left(\theta\:,{P}_{c}\right)={c}_{\begin{array}{c}{1}_{j}\\\:\:\end{array}}{{{P}_{c}}_{j}}^{2}+{c}_{{2}_{j}}{P}_{{c}_{j}}(1-{{\theta\:}_{j}}_{\:})$$

Where$$\:\:{\theta\:}_{j}$$ represents the customer $$\:j\:$$ type and its value describe his willingness to participate in the DRP; the most willing have $$\:\theta\:=1,$$ and the least have $$\:\theta\:=0$$$$\:;{P}_{{c}_{j}}$$ is the amount of power reduction; $$\:{c}_{{1}_{j}}$$and $$\:{c}_{{2}_{j}}$$are customers $$\:j\:$$cost coefficients.

The benefit of $\:j\:$customer ($\:{B}_{1,j}$) who participates in DRP can be calculated as:

4 $$\:{B}_{1,j}(\theta\:,{F}_{j},{P}_{c})={F}_{j}-Cos{t}_{j}^{cust}\left(\theta\:,{P}_{c}\right)$$

Where $\:{F}_{j}$ is customer $\:j$ incentive payment.

The MMG benefit from the participation of customer $\:j\:$in DRP is calculated based on the cost of power interruption coefficient $\:\left({\uplambda\:}\right)$ as^45^:

5 $$\:{B}_{2}\left(\theta\:,\lambda\:,{P}_{{c}_{j,t}}\right)={\lambda\:}_{j}{\:P}_{{c}_{j,t}}-{F}_{j,t}\:\:\:\:\:\:\:\:\:\:\:\:\:\:$$

Then the MMG total benefit is expressed as:

6 $$\:{\text{B}}_{0}=\sum\:_{\text{j}=1}^{\text{J}}\sum\:_{t=1}^{T}{{\uplambda\:}}_{\text{j},\text{t}}{\:\text{P}}_{{\text{c}}_{\text{j},\text{t}}}-{\text{F}}_{\text{j},\text{t}}\:\:\:\:\:\:\:\:\:\:\:\:\:\:\:\:\:$$

## Techno-economic environmental multi-layer multi-objective energy management

**Fig. 2:**
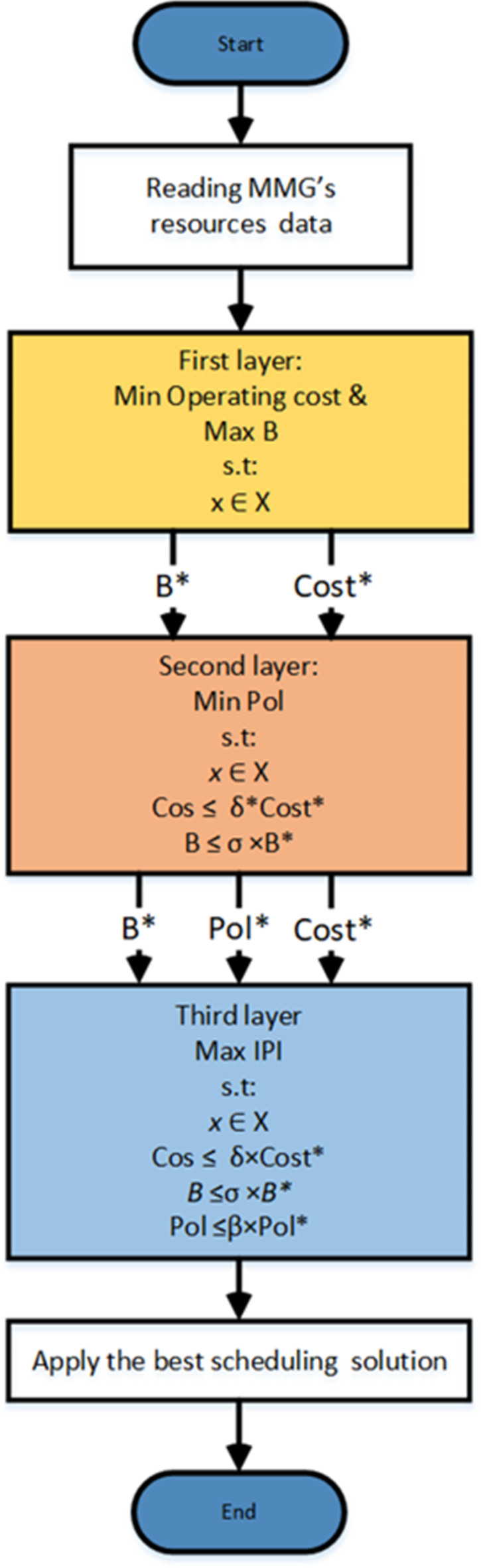
Flowchart of the proposed multi-layer hybrid technique.

The proposed multi-objective framework for Energy Management System (EMS) in MMG offers a unique approach for decision-makers, as it simultaneously considers techno-economic environmental aspects, and the integration of DRP in EMS. The model comprises of three layers with four objectives: operating cost (generation and power transaction costs), MMG benefit, greenhouse gas emissions, and MMG independence from the UG. Various techniques can be employed to optimize multi-objectives, such as the weighted sum technique, normalized weighted sum technique, Pareto principle with fuzzy logic techniques, epsilon constraint technique, compromised programming technique, and lexicography technique. In this paper, the MOO problem is transformed into a multi-layer optimization framework, utilizing a hybrid ε-lexicography-weighted sum technique. Unlike the compromised programming and weighted-sum techniques, the proposed technique eliminates the need for the normalization process of the objective functions. Furthermore, this hybrid technique reduces the need for multi-attribute decision-making for solution selection and ranking, as in the Pareto principle with fuzzy logic techniques and the epsilon constraint technique. In the proposed hybrid technique, the objectives are ranked based on the decision-makers’ perspective. Each layer is responsible for achieving one or more objectives, and separating the objectives into different layers eliminates the need for normalization of the objectives and the need to adjust the weighting factors for all objectives. Figure 2 demonstrates the flowchart of the proposed MLMO hybrid technique. As illustrated in Fig. 2, the data of the resources of every SMG, such as conventional generation data, renewable generation data, customers’ data, and DRP data, are imported at the CO point. These data are used as input for each optimization layer. In the first layer, the CO uses the MMG data in solveing the EM problem for the optimal scheduling of the resources, which is a MOO problem. The optimal operation is to reduce the operating cost, which includes the cost of power generation from the DGs and the cost of power transactions with the UG. By the end of this layer, the minimum operating cost ($\:cos{t}^{*}$) and the maximum benefit ($\:{B}_{0}^{*}$) are calculated and used as input for the following layers. Then, in the second layer, the resources’ primary scheduling is alerted to improve the greenhouse gas emissions; the minimization of the emissions is performed with keeping the operating cost and the benefit within acceptable ranges. Then the minimum operating emissions ($\:Po{l}^{*}$) is determined and its value used as input data to the last layer. In the third layer,$\:\:cos{t}^{*}$, $\:{B}^{*}$, and $\:Po{l}^{*}$ are used as a constraints to keep cost, benefit and the emissions at an acceptable range. Then the optimal scheduling of the independence from the UG is maximized, as the EMS tries to supply the loads mainly from local generation resources.

### Economic layer: first optimization layer

The objective of this layer is to achieve a multi-objective economic operation. The optimal operation of the first objective is to minimize the cost of local resources generation and the cost of power transaction with the UG as follows:

7 $$\:\text{M}\text{i}\text{n}\text{m}\text{i}\text{z}\text{e}\:{\text{F}}_{1}\left(\text{x}\right)=\text{M}\text{i}\text{n}\:(\:cost=\sum\:_{t=1}^{T}\sum\:_{i=1}^{I}{cost}_{i}^{DG}\left({P}_{{i}_{t}}\:\right)+\sum\:_{t=1}^{T}cos{t}_{t}^{UG}\left({P}_{U{G}_{t}}\:\right))$$

Where $\:I$ is the total number of conventional DGs, and $\:T$ is the total time horizon (h).

The second objective is to maximize the CO benefit through the DRP as:

8 $$\:\text{M}\text{a}\text{x}\text{i}\text{m}\text{i}\text{z}\text{e}\:\:{\text{F}}_{2}\left(x\right)=\text{M}\text{a}\text{x}\:{(B}_{0}={\sum\:}_{\text{t}=1}^{\text{T}}\sum\:_{j=1}^{J}{\lambda\:}_{j}{\:P}_{{c}_{j}}-{F}_{j}\:)$$

In this layer, an internal weighting-sum technique is used so that the mathematical expression of the objective function is:.

9 $$\:\text{F}\left(\text{x}\right)=\text{M}\text{i}\text{n}\left({\:w}_{1}\left[\sum\:_{t=1}^{T}\sum\:_{i=1}^{I}{cost}_{i}^{DG}\left({P}_{{i}_{t}}\:\right)+\sum\:_{t=1}^{T}Cos{t}_{t}^{UG}\left({P}_{U{G}_{t}}\:\right)\right]{+\text{w}}_{2}\:\left[{\sum\:}_{\text{t}=1}^{\text{T}}\sum\:_{j=1}^{J}{{F}_{j}-\lambda\:}_{j}{\:P}_{{c}_{j}}\:)\right]\right)$$

Where the weighting factors $\:\:{\:w}_{1},{w}_{2}$ should satisfy $\:{w}_{1}+{w}_{2}=1$.

The EM problem is solved, and the optimal value for objectives cost $\:\left(cos{t}^{*}\right)$, and benefit $\:\left({B}^{*}\right)$ are determined and imported to the following layers to be integrated into their optimization process.

### Environmental layer: second optimization layer

Once the optimal schedule for the first layer has been determined and its data imported, the second layer begins optimizing its objectives while also ensuring that the objectives from the previous layer remain within the acceptable range set by the CO. The second layer is focused on minimizing the greenhouse gas emissions resulting from the generation of distributed generators (DGs) and from the grid. The objective function for this layer is mathematically formulated as follows^46^:

10 $$\begin{aligned}\text{M}\text{i}\text{n}\text{i}\text{m}\text{i}\text{z}\text{e}\:\text{F}_{3}\left(x\right) =\text{M}\text{i}\text{n}&(\text{P}\text{o}\text{l}=\mathop{\sum}\nolimits_{\text{t}=1}^{\text{T}}\mathop{\sum}\nolimits_{i=1}^{I}{\mathop{\sum}\nolimits_{\text{k}} } {\gamma}_{\text{i}_{\text{k}}} P_{i_{t}}+\mathop{\sum}\nolimits_{t=1}^{T}{\mathop{\sum}\nolimits_{\text{k}}} {\gamma}_{\text{UG}_{\rm k}} P_{\text{UG}_{t}}) \\&\qquad \qquad Subject\, to: \\&\quad \:cost\le\:\delta\:\times\:cos{t}^{*} \\&\qquad \quad \:{B}_{O}\ge\:\sigma\:\times\:{{B}^{*}}_{o}\end{aligned}$$

Where$\:\:k$ is the type of pollutant (CO_2_, SO_2_, NOx); $\:{{\upgamma\:}}_{{\text{i}}_{\text{k}}}$ is the pollutant emission coefficient (g/kw) of the $\:{i}^{th}$ DG; $\:{{\upgamma\:}}_{{\text{U}\text{G}}_{\text{k}}}$ is the pollutant emission coefficient (g/kw) of the UG.

In Eq. ‎(12), $$\:\delta\:$$$$\:\ge\:1$$and $$\:\sigma\:\le\:1$$ are constants. The value of this constant plays an important role in this layer scheduling. Setting the value of $$\:\delta\:=1$$ means that the emissions should be minimized with considering keeping the operating cost at its reference value (minimum value) or lower, but this will limit the chance for minimization of the pollutant; other values of $$\:\delta\:$$ can help in minimizing the emissions, but this may cause an increase in the operating cost. The same is valid for the value of $$\:\sigma\:$$ with the pollutant emissions. In other words, the values of $$\:\delta\:$$ and $$\:\sigma\:$$ are a controlling parameters in the search space, and there is no precise technique for setting their value; in this paper, $$\:\delta\:=1.05$$ and $$\:\sigma\:=0.95$$ are choosen. At the end point of this optimization layer, the optimal value of pollutant ($$\:Po{l}^{*}$$) is determined and imported to the following layer.

### Technical layer: third optimization layer

As stated before, the MMG is connected to the UG, where the energy can be transacted between MMG and the UG. To enhance the dependability of the MMG, this layer will alert the scheduling from the previous layer of maximizing the independence performance index (IPI). The objective function of this optimization layer can be mathematically expressed as follows:

11 $$\begin{aligned}\text{M}\text{a}\text{x}\text{i}\text{m}\text{i}\text{z}\text{e}\:{f}_{4}\left(x\right)=&\text{M}\text{a}\text{x}(IPI=1-\frac{\sum\:_{t=1}^{T}\left|{P}_{{UG}_{t}}\right|}{{\sum\:}_{t}^{T}{L}_{t}-{\sum\:}_{t=1}^{T}\sum\:_{j=1}^{J}{\:P}_{{{c}_{j}}_{t}}\:\:}\\&\qquad \ Subject\,to:\\& cost\le\:\delta\:\times\:cos{t}^{*}\\&\qquad \quad {B}_{0}\ge\:\sigma\:\times\:{B}_{0}^{*}\\&\qquad \ Pol\le\:\beta\:\times\:Po{l}^{*}\end{aligned}$$

Where $\:{L}_{t}$ is the total demand at any time interval $\:t$.

In this layer, the dependent on the UG is minimized by reducing the power exchange between MMG and UG and depending on the local generation mainly to feed the total load demand with considering the DRP. The maximization of $\:IPI$ is performed while maintaining$\:cost$, $\:B,$ and $\:Pol\:$ to be within the acceptable range limited by constants $\:\delta\:,\:\sigma\:$, and $\:\beta\:$. Where $\:B$ is constant, which has the role of maintaining the pollutant emissions within the pre-specified range; in this paper, $\:\beta\:=1$ is selected.

### Constraints

The MLMO optimization problem is solved by considering some constraints as:

- ***Energy balance constraints***
  At any time interval $\:t,\:$the total generation with the power transacted with the UG should equal to the total demand as^25^:
  12 $$\:\sum\:_{i=1}^{I}{P}_{{i}_{t}}+{P}_{{UG}_{t}}+{{P}_{WT}}_{t}+{{P}_{PV}}_{t}={L}_{t}-\sum\:_{j=1}^{J}{{P}_{c}}_{j,t}\:\:\:\:\:\:\:\:\:\:\:\:\:\:\:\:\:\:$$
- ***Conventional generators constraints***
  13 $$\:P_{{i}_{min}}\le\:P_{{i}_{t}}\le\:P_{{i}_{max}}$$
  14 $$\:{-RD}_{i}\le\:{P}_{{i}_{t+1}}-{P}_{{i}_{t}}\le\:{RU}_{i}$$

For generator $\:i$, $\:{P}_{{i}_{max}}$ and $\:{P}_{{i}_{min}}$are the maximum and minimum generation limits, $\:{RU}_{i}\:$ and $\:{RD}_{i}$ are the ramp-up and down maximum rates^25^.

- ***Utility Grid Constraints***
  Energy transaction with the main grid at any time interval $\:t$ is limited with the maximum permissible rates $\:{P}_{{UG}_{max}}$ as:
  15 $$\:{P}_{{UG}_{max}}\le\:{P}_{{UG}_{t}}\le\:{P}_{{UG}_{max}}$$
- ***Incentive Demand Response constraints***

Based on the IDRP discussed in section ‎0, the Customer’s benefit in Eq. ‎(4) is extended to be for the whole time horizon $\:T$, so customer benefit can be expressed as:

16 $$\:\sum\:_{t=1}^{T}{F}_{j,t}-Cos{t}_{j,t\:}^{cust}\ge\:0$$

The curtailment for each customer is also subjected to a constraint expressed as:

17 $$\:\sum\:_{j=1}^{J}{{P}_{c}}_{j,t}\le\:{CL}_{j}$$

Where $\:C{L}_{j}$ is the $\:{j}^{th}$ customer daily curtailment limit.

Constraint in Eq. ‎(8) is for the total budget that CO can pay daily^25^.

18 $$\:\sum\:_{t=1}^{T}\sum\:_{j=1}^{J}{B}_{j,t}\le\:UBL$$

## Uncertainty modeling

In the presence of random or uncertain input variables, the EMS should treat the EM problem as probabilistic rather than deterministic. The random nature of solar irradiation and wind speed also makes the generated power from PVs and WTs random. In addition, the load demand is considered a random variable because of forecasting errors, load variation, or unexpected disturbance^47^. A statistical description of the input random variable is required to assess the effect of the random variable. Consider the uncertain nature of the PV power, WT power, energy cost, and load demand.

### Generating PDF for uncertainty variables

Solar irradiance ($\:{G}_{PV}$)is assumed to have a lognormal distribution, then the probability density function (PDF) can be articulated as^48^:

19 $$\:{f}_{{G}_{PV}}\left({G}_{PV}\right)=\frac{1}{{G}_{PV}{\sigma\:}_{PV}\sqrt{2\pi\:}}.\:{e}^{-\left({\text{l}\text{n}(G}_{PV}\right)-{\mu\:}_{PV}{\:)}^{2}/2{\sigma\:}_{PV}^{2}}\:$$

And the generated power from PV is:

20 $$\:{P}_{PV}\left({G}_{PV}\right)=\left\{\begin{array}{c}{P}_{P{V}_{r}}\left(\frac{{G}_{PV}}{{G}_{std}\times\:{X}_{c}}\right)\:\:\:\:\:\:\:\:\:\:for\:0<{G}_{PV}\le\:{X}_{c}\:\\\:{P}_{P{V}_{r}}\left(\frac{{G}_{PV}}{{G}_{std}}\right)\:\:\:\:\:\:\:\:\:\:\:\:\:\:\:\:\:for\:{G}_{PV}\ge\:{X}_{c}\:\:\:\:\:\:\:\:\:\:\:\:\end{array}\right.\:$$

Where $\:{{\text{P}}_{\text{P}\text{V}}}_{\text{r}}$ is the rated power of PV unit, $\:{\text{X}}_{\text{c}}$ is the irradiance constant (120 = w/m^2^), $\:{\text{G}}_{\text{s}\text{t}\text{d}}$ is the standard solar irradiation (800 = w/m^2^).

The probability of Wind speed $\:\text{v}$ can be expressed by Weibull PDF as^49^:

21 $$\:{f}_{{v}_{WT}}\left(v\right)=\left(\frac{k}{c}\right){\left(\frac{v}{c}\right)}^{k-1}.\:{e}^{-{\left(\frac{v}{c}\right)}^{k}}\:, \:k={\left(\frac{{\sigma\:}_{WT}}{{\mu\:}_{wt}}\right)}^{-1.086}, \:k=\frac{{\mu\:}_{WT}}{{\Gamma\:}\left(1+\frac{1}{k}\right)}$$

Where $\:\text{k}$ and $\:\text{c}$ are the shape and scale index parameters, $\:{{\upsigma\:}}_{\text{W}\text{T}}$ and $\:{{\upmu\:}}_{\text{W}\text{T}}\:$are standard deviation and mean values.

And the generated power can be computed as:

22 $$\:{P}_{WT\:}=\left\{\begin{array}{c}0\:\:\:\:\:\:\:\:\:\:\:\\\:\frac{\begin{array}{c}v\\\:{v}_{WT}^{2}-{v}_{in}^{2}\end{array}}{{v}_{nom}^{2}-{v}_{in}^{2}}.\\\:{P}_{W{T}_{r}}\end{array}\right.{P}_{W{T}_{r}}\:\:\:\:\:\:\:\:\:\genfrac{}{}{0pt}{}{\begin{array}{c}v\le\:{v}_{in}\:and\:\:v\ge\:{v}_{out}\\\:\:\end{array}}{\begin{array}{c}{v}_{in}\le\:v\le\:{v}_{r}\:\:\\\:\:\\\:{v}_{r}<v\le\:{v}_{out}\end{array}}\:$$

Where $\:{\text{P}}_{\text{W}{\text{T}}_{\text{r}}}$ is the rated power of wind turbine, $\:{\text{v}}_{\text{i}\text{n}}$ and $\:{\text{v}}_{\text{o}\text{u}\text{t}}$ are the cut in and cut out wind speed, $\:{\text{v}}_{\text{r}}$ is the rated wind speed.

The uncertainties of load and energy prices are modeled using normal PDF^49^. So for any random variable ${\text{G}}_{\text{i}}$ the PDF can be expressed as:

23 $$\:{f}_{{G}_{i}}\left({G}_{i}\right)=\frac{1}{\sigma\:\sqrt{2\pi\:}}.\:{e}^{-({G}_{i}-\mu\:{)}^{2}/2{\sigma\:}^{2}}\:$$

Where $\:{\upmu\:}$ and $\:{\upsigma\:}$ are the mean and standard division (SD) values respectively for variable $\:{\text{G}}_{\text{i}}$.

### Point Estimation Method (PEM)

Compared to other approaches used for modeling uncertainty, PEM has proven to be an effective method for studying power systems. PEM reduces the computational and gives a high degree of accurate results^50,51^. To get a statistical characterization approximation of any output variable $\:\text{z}$, this estimation method concentrates the data of the first few moments for the random input variable (RIV) $\:{\text{p}}_{\text{l}}$ on $\:\text{k}=\text{2,3},5,\dots\:$ points. i.e. $\text{Z}=\text{F}({\text{p}}_{1},{\text{p}}_{2},{\text{p}}_{3},\dots\:.,{\text{p}}_{\text{m}})$ where ${\text{p}}_{1},{\text{p}}_{2},{\text{p}}_{\text{m}}$ are the first, second and mth random input variable. The central moment of the output variable $\:\text{y}$ is evaluated number of times based on the adopted estimation scheme ($\:2\text{m},2\text{m}+\text{1,4}\text{m}+1)$

## 2 M + PEM

A composition locations $\:{\:p}_{L,k}$and wights $\:{w}_{L,k}$ define the $\:{k}^{th}$ concentration of the IRV $\:{p}_{l}$. In this scheme, there are three concentration points on the standard locations for any $\:{p}_{l}$ IRV; the standard locations are calculated as:

24 $$\:{\xi\:}_{L,k}=\frac{{\lambda\:}_{L,3}}{2}+(-1{)}^{3-k}.\sqrt{{\lambda\:}_{L,4}-\frac{3}{4}}{{\lambda\:}^{2}}_{L,3}\:\:\:\:\:k=\text{1,2}\:and\:{\xi\:}_{L,3}=0$$

With $\:{\xi\:}_{t,3}$ represents the mean value location,$\:\:{\lambda\:}_{t,3}$ is the skewness (third moment), and $\:{\lambda\:}_{t,4}$ is the kurtosis of the IRV.

Then the locations of the variables can be computed as:

25 $$\:{\:p}_{L,k}\:={\mu\:}_{{p}_{L}}+{\xi\:}_{L,k}.\:{\sigma\:}_{{p}_{t}}\:\:\:\:\:\:k=\text{1,2},3$$

Weights for the standard locations are:

26 $$\:{w}_{L,k}=\frac{{(-1)}^{3-k}}{{\xi\:}_{L,k}({\xi\:}_{L,1}-{\xi\:}_{L,2})}\:\:\:\:\:\:\:k=\text{1,2}$$

27 $$\:{w}_{L,3}=\frac{1}{m}-\frac{1}{{\lambda\:}_{L,4}-{{\lambda\:}^{2}}_{L,3}}$$

Moreover, in order to mitigate the probabilistic nature of optimization algorithms, the problem is solved for a variety of runs, and the average value is then calculated. Then, the row moments’ vector for the output variable $\:E\left(Z\right)$ is computed based on the vector $\:Z(l,k)$ as:

28 $$\:E\left({Z}^{j}\right)\approx\:E\left({Z}^{j}\right)+\sum\:_{l=1}^{2}{w}_{l,k}.{\left[F\left(Z({p}_{l,k}\right)\right]}^{j}\:\:\:\:\:\:$$

If we set $\:k=3$ in Eq. ‎(25) and from Eq. ‎(24) $\:{\xi\:}_{L,3}=0$ this yield the standard location $\:\:{\:p}_{L,k}={\mu\:}_{{p}_{L}}$ or the mean value. This is valid for all variables. So, the output function is evaluated only twice and one time for the mean value of all variables ( $\:{\mu\:}_{{p}_{1}},{\mu\:}_{{p}_{2}},\dots\:,{\mu\:}_{{p}_{t}},\dots\:,{\mu\:}_{{p}_{m}}$). And so the corresponding weights of this location can be expressed as:

29 $$\:{w}_{0}=1-\sum\:_{L=1}^{m}\frac{1}{{\lambda\:}_{L,4}-{{\lambda\:}^{2}}_{L,3}}$$

The row moments’ vector for the output variable $\:E\left(Z\right)$ is computed based on the vector $\:Z(l,k)$ as:

30 $$\:E\left({Z}^{j}\right)\approx\:E\left({Z}^{j}\right)+{w}_{0}.{\left[F\left(Z({P}_{\mu\:}\right)\right]}^{j}$$

Then the mean and standard deviation of the output variable are obtained as:

31 $$\:{\mu\:}_{z}=E\left(Z\right)\:\:\:\:\:\:\:\:\:$$

32 $$\:{\sigma\:}_{z}=\sqrt{E\left({Z}^{2}\right)-{{\mu\:}^{2}}_{Z}\:}\:\:\:\:$$

The PDF of the output variable is then computed using Gram–Charlier series based on $\:{\mu\:}_{Z}$ and $\:{\sigma\:}_{Z}$^52^.

**Fig. 3:**
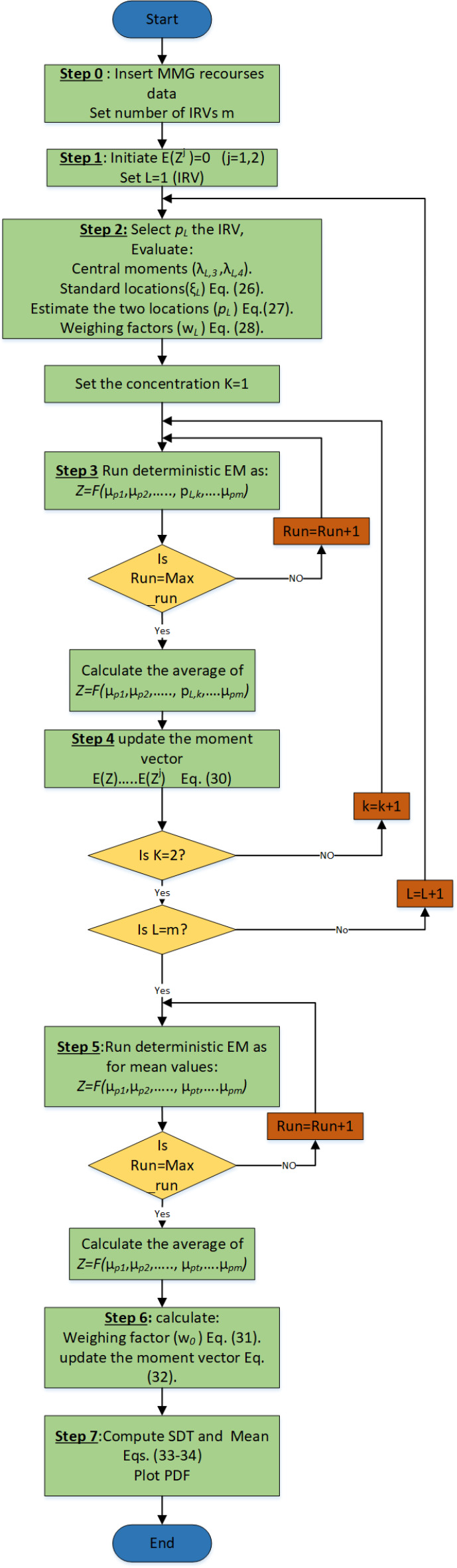
Flowchart of 2m + 1 PEM for probabilistic EM.

The flowchart of the 2 m + 1 PEM for considering the uncertainties existing in the MMG is shown in Fig. 3.

## Solution method

### Equilibrium Optimizer (EO)

The Equilibrium Optimizer is a recently created algorithm that is based on principles of physics. Its development draws inspiration from control volume mass balancing models, which are used to calculate both equilibrium and dynamic states^53,54^. This algorithm estimates the mass entering and leaving a control volume based on the mass balance equations. The following phases describe the EO algorithm mathematical model:

- **Initialization**: Similar to other metaheuristic algorithms, EO starts with initializing the population $\:\left(N\right)$of particles with uniform random distribution based on the upper boundary $\:\left({X}_{UB}\right)$ and the lower boundary $\:{X}_{LB}$ as:
  33 $$\:{\text{X}}_{i}^{\text{i}\text{n}\text{i}\text{t}\text{i}\text{a}\text{l}}={\text{X}}_{\text{L}\text{B}}+\text{r}\text{a}\text{n}\text{d}\text{*}\left({\text{X}}_{\text{U}\text{B}}-{\text{X}}_{\text{L}\text{B}}\right)\:\:\:\text{i}=\text{1,2},\dots\:\dots\:,\text{N}$$
- **Equilibrium**: The primary objective of the EO algorithm is to identify the global optimum point, or equilibrium point. To achieve this, the algorithm evaluates several equilibrium candidates in order to determine the optimal search pattern. These candidates consist of the top four particles that have been identified throughout the optimization process, along with a fifth candidate whose concentration is the average of the first four. Together, these five candidates form what is referred to as the equilibrium pool.
  34 $$\:{\overrightarrow{X}}_{eq}^{pool}={{\{\overrightarrow{X}}_{eq}}^{\left(1\right)},{{\overrightarrow{X}}_{eq}}^{\left(2\right)},{{\overrightarrow{X}}_{eq}}^{\left(3\right)},{{\overrightarrow{X}}_{eq}}^{\left(4\right)},{{\overrightarrow{X}}_{eq}}^{\left(avg\right)}\}$$
- **Update stage**: In each iteration, the updating of the concentration of particles is achieved based on a random selection from candidates for equilibrium with equal probability. This will continue until the completion of the optimization procedure. There are two terms that affect the updating process as follows:.
- **Exponential term**: this term help in enhancing the balancing in exploration and exploitation phases, and it can be calculated as:
  35 $$\:\overrightarrow{F}={e}^{-{\lambda\:}_{e}({t}_{iter}-{t}_{o})}$$

Where $\:{\lambda\:}_{e}$ is a random number in the interval of [0,1], and $\:{t}_{{\:}_{iter}}$ can be calculated as a function of iterations as:

36 $$\:{t}_{iter}={\left(1-\frac{Iter}{Ite{r}_{\text{max}\:}}\right)}^{{a}_{2}*\left(\frac{Iter}{Ite{r}_{\text{max}\:}}\right)}$$

Where $\:Iter$ and $\:Ite{r}_{\text{max}\:}$ are the current and maximum number of iterations, $\:{a}_{2}$is a constant number controlling the exploitation process; in this paper, it has a value of 1.

And $\:{t}_{0}$ is calculated as:

37 $$\:\overrightarrow{{t}_{0}}={1\frac{1}{\overrightarrow{{\lambda\:}_{e}}}\left(-{a}_{1}sign(\overrightarrow{r}-0.5\right)[1-{e}^{-\overrightarrow{{\lambda\:}_{e}}{t}_{iter}}])+{t}_{iter}}^{\:}$$

Where $\:{a}_{1}$ is a constant control the exploration process and has a value of 2; the term $\:sign(\overrightarrow{r}-0.5)$ affect the direction of exploration and exploitation.

- Generation Rate: this term is to enhance the exploitation phase, it can be described as a first-order exponential decay as:
  38 $$\:\overrightarrow{GR}={\overrightarrow{G{R}_{0}}{e}^{-\overrightarrow{k}({t}_{iter}-{t}_{0})}}^{\:}$$

Where $\:k$ is a random decay variable, to limit the randomness of the search pattern $\:k={\lambda\:}_{e}$  is selected so eq. ‎(38) will be as:

39 $$\:\overrightarrow{GR}={\overrightarrow{G{R}_{0}}{e}^{-\overrightarrow{{\lambda\:}_{e}}({t}_{iter}-{t}_{0})}}^{\:}=\overrightarrow{G{R}_{0}}\overrightarrow{F}$$

Where $\:G{R}_{0}$ is the initial generation expressed as:

40 $$\:\overrightarrow{G{R}_{0}}=\overrightarrow{G{RCP}_{\:}}\times\:({X}_{eq}-\overrightarrow{{\lambda\:}_{e}}\overrightarrow{X})\:$$

Where $\:G{RCP}_{\:}$ is the generation rate control parameter which defines the possibility for the contribution of the generation term in the updating process and its value depend on the generation rate probability $\:G{RP}_{\:}$ ($\:G{RP}_{\:}$ =0.5 is selected for good balance between exploitation and exploration) as follow:

41 $$\:\overrightarrow{G{RCP}_{\:}}=\left\{\begin{array}{c}0.5{r}_{1}\:\:\:\:\:\:{r}_{2}\ge\:GRP\\\:0\:\:\:\:\:\:\:\:\:\:\:{r}_{2}<GRP\end{array}\:\:\right.\:\:$$

Where $\:{r}_{1},{r}_{2}$ are random variables in the interval [0,1].

So the candidate updated as:

42 $$\:\overrightarrow{X}={\overrightarrow{X}}_{eq}+\left(\overrightarrow{X}-{\overrightarrow{X}}_{eq}\right).\overrightarrow{F}+\overrightarrow{\frac{GR}{{\overrightarrow{\lambda\:}}_{e}V}}(1-\overrightarrow{F})\:$$

Where $\:V$ is a unit.

### Enhanced equilibrium Optimizer

The EEO algorithm has been designed to address the limitations of the original EO algorithm^55^, including issues with local minima and difficulties with balancing exploration and exploitation in high-dimensional problems. To prevent the algorithm from becoming stuck in local optima and to improve convergence speed, the EEO algorithm incorporates a hybridization of the EO algorithm and the Sine Cosine algorithm (SCA) to update the particles’ concentration. SCA is considered a simple and effective dynamic algorithm. One of the main advantages of SCA is its ability to maintain balance between exploration and exploitation in the search space. Sine and Cosine functions pay a pivotal role in the updating process for enhancing the convergence characteristics of the original EO towards the equilibrium state.

This updating process is expressed using a random variable within the range of $\:{r}_{4}\in\:$ [0,1], as follows:

43 $$\:\overrightarrow{X}=\:\left\{\begin{array}{c}{\overrightarrow{X}}_{eq}+\left(\overrightarrow{X}-{\overrightarrow{X}}_{eq}\right).\overrightarrow{F}+\overrightarrow{\frac{GR}{\overrightarrow{\lambda\:}V}}\left(1-\overrightarrow{F}\right)\:\:\:\:\:if\:{r}_{4}>0.5\\\:u\:\:\:\:\:\:\:\:\:\:\:\:\:\:\:\:\:\:\:\:\:\:\:\:\:\:\:\:\:\:\:\:\:\:\:\:\:\:\:\:\:\:\:\:\:\:\:\:\:\:\:\:\:\:else\end{array}\:\:\:\:\:\right.\:\:$$

And $\:u$ is obtained as follows:

44 $$\:u=\:\left\{\begin{array}{c}\left(\overrightarrow{X}-{\overrightarrow{X}}_{eq}\right)+{R}_{1}\text{sin}\left({R}_{2}\right)\times\:\left({\overrightarrow{X}}_{eq}-\overrightarrow{X}\right)\:\:\:\:\:if\:{r}_{5}>0.5\\\:\left(\overrightarrow{X}-{\overrightarrow{X}}_{eq}\right)+{R}_{1}\text{cos}\left({R}_{2}\right)\times\:\left({\overrightarrow{X}}_{eq}-\overrightarrow{X}\right)\:\:\:\:\:\:\:\:\:\:\:\:\:\:\:\:\:else\end{array}\:\:\:\:\:\right.\:\:$$

And $\:{R}_{1}and\:{R}_{2}$ are calculated as:

45 $$\:{R}_{1}=0.01\times\:{t}_{2}$$

46 $$\:{R}_{2}=\pi\:\times\:rand$$

$\:{t}_{2}$ value decreases with the increase of the iteration as:

47 $$\:{t}_{2}={\left(1-\frac{Iter}{Ite{r}_{\text{max}\:}}\right)e}^{{a}_{2}*\left(\frac{Iter}{Ite{r}_{\text{max}\:}}\right)}$$

## Simulation results

**Fig. 4:**
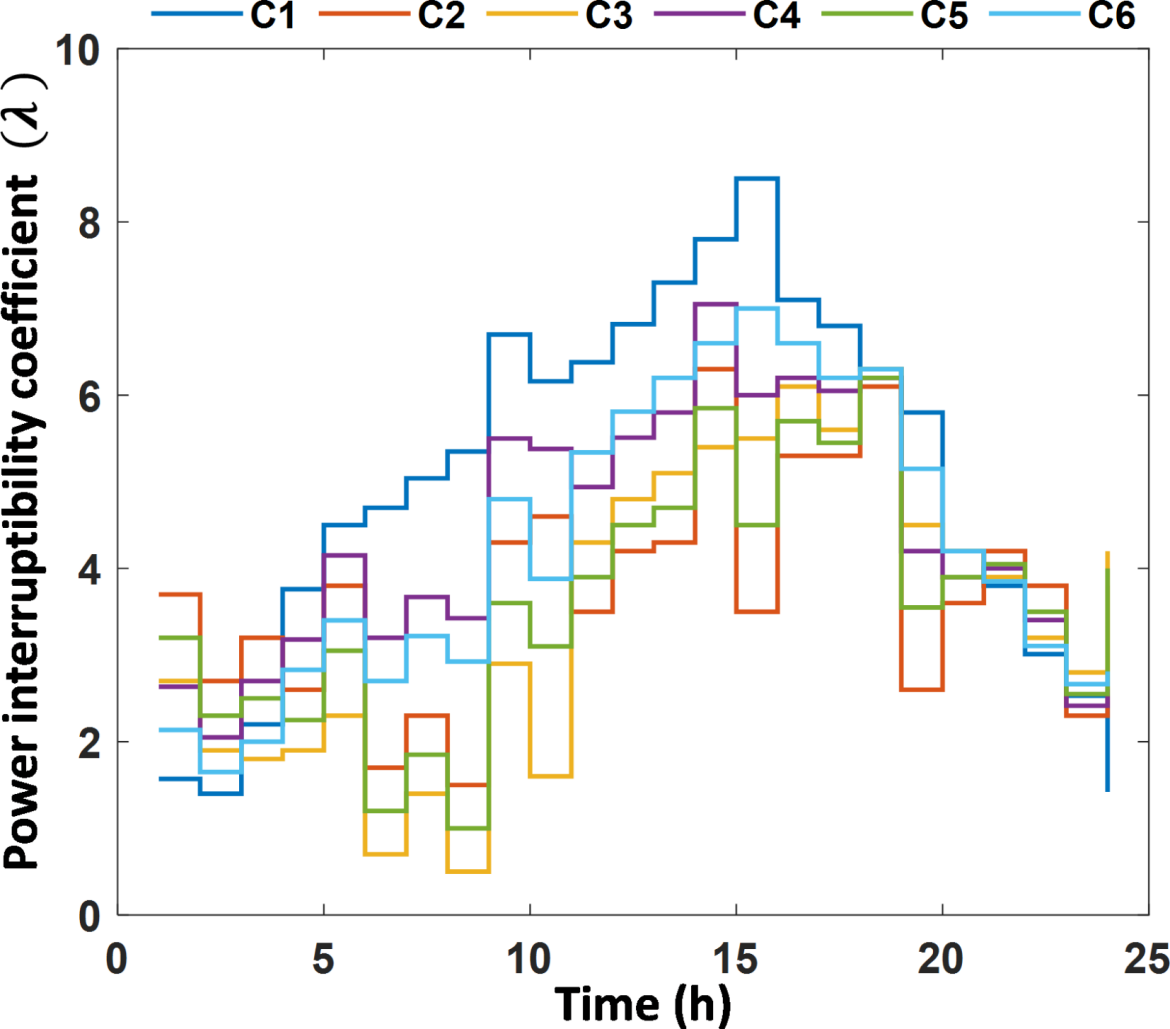
Cost of power interruptibility coefficient.

To validate its performance, the MLMO with EEO is applied to solve the EM problem on a MMG test system. The MMG model that is used for this purpose comprises of three SMGs that are interconnected with each other. Each SMG is equipped with different resources and customers (Cs), and their specific characteristics are detailed in Table 2. The parameters for DGs used in MMG are presented in Table 3. The maximum forecasted PV power, Wind power, and total initial demand are shown in Table 4^25,56^. It is assumed that the solar panels and wind turbines have the same rating for PV_1_ and PV_2_ and WT_1_ and WT_2_, respectively, all customers are assumed to participate actively through the DRP; their data, including customer type, cost coefficients and the curtailment level, are presented in Table 5. The customers’ cost of power interruption coefficient is shown in Fig. 4. Three different simulation case studies for the propose MMG architecture, as depicted in Fig. 1, are conducted based on MATLAB 2021b on a PC with 2.9-GHz i7 with 8-GB of RAM. The EEO algorithm is used to solve the EM problem and the details of the case studies as following:

- Case I: Single-layer bi-objective EMS is applied, where the DRP is integrated, and the objectives are minimizing the operating cost while maximizing the CO benefit from the DRP.
- Case II: Multi-layer Multi-objective EM problem; in this Case, four objectives are considered. The operating cost, CO benefit, emissions, and independency from the UG are simultaneously optimized.
- Case III: based on 2 m + 1 PEM a probabilistic simulation considering the uncertainties in renewable generation, load, and energy prices.

**Table 2:** Microgrids characteristics.

	SMG 1	SMG 2	SMG 3
Diesel generators	DG_1_, DG_3_, DG_3_	×	DG_4_,DG_5_,DG_6_
Wind	WT_1_	WT_2_	×
PV	PV_1_	PV_2_	PV_3_
Customers	C_1_,C_2_,C_3_	C_4_	C_5_,C_6_

**Table 3:** Diesel Generators parameters.

DG_i_	a_i_($/kW^2^h)	b_i_($/KW h)	*P*_i, min_ (KW)	*P*_i, max_(KW)	DR_i_ (KW/h)	UR_i_ (KW/h)
*DG*_1_,*DG*_4_	0.06	0.5	0	4	3	3
*DG*_*2*_,*DG*_*5*_	0.03	0.25	0	6	5	5
*DG*_*3*_,*DG*_*6*_	0.04	0.3	0	9	8	8

**Table 4:** Hourly solar power, Wind Power, and initial load demand data.

Time (h)	PV_1_,PV_2_ (kw)^25^	PV_3_(kw)^56^	WT_1_,WT_2_ (kw)^25^	Load demand (kw)
t = 1	0	0	7.56	62.07
t = 2	0	0	7.5	61.23
t = 3	0	0	8.25	60.78
t = 4	0	0	8.48	60.45
t = 5	0	0	8.48	60.78
t = 6	0	0	9.42	62.60
t = 7	0	0	9.82	64.29
t = 8	7.99	3.19	10.35	66.50
t = 9	10.56	4.22	10.88	73.18
t = 10	13.61	5.44	11.01	74.74
t = 11	14.97	5.99	10.94	78.06
t = 12	15	6	10.68	80.28
t = 13	14.78	5.91	10.42	77.36
t = 14	14.59	5.84	10.15	81.32
t = 15	13.56	5.42	9.67	82.10
t = 16	11.83	4.73	8.98	81.26
t = 17	10.17	4.07	8.37	79.37
t = 18	7.66	3.06	7.61	78.14
t = 19	0	0	6.7	75.33
t = 20	0	0	5.72	70.98
t = 21	0	0	7.21	66.50
t = 22	0	0	7.75	63.96
t = 23	0	0	7.88	63.38
t = 24	0	0	7.69	62.40

**Table 5:** Customers Data.

C_j_	$\:\varvec{\uptheta\:}$	K_1,j_	K_2,j_	$\:{\mathbf{C}\mathbf{M}}_{\mathbf{j}}$(KW)
**C**_**1**_	0	1.079	1.32	30
**C**_**2**_	0.45	1.378	1.36	35
**C**_**3**_	0.8	1.847	1.64	40
**C**_**4**_	0.2	1.079	1.32	30
**C**_**5**_	0.55	1.378	1.36	35
**C**_**6**_	0.9	1.847	1.64	40

- **Case I: Deterministic Single-Stage EM**

**Fig. 5:**
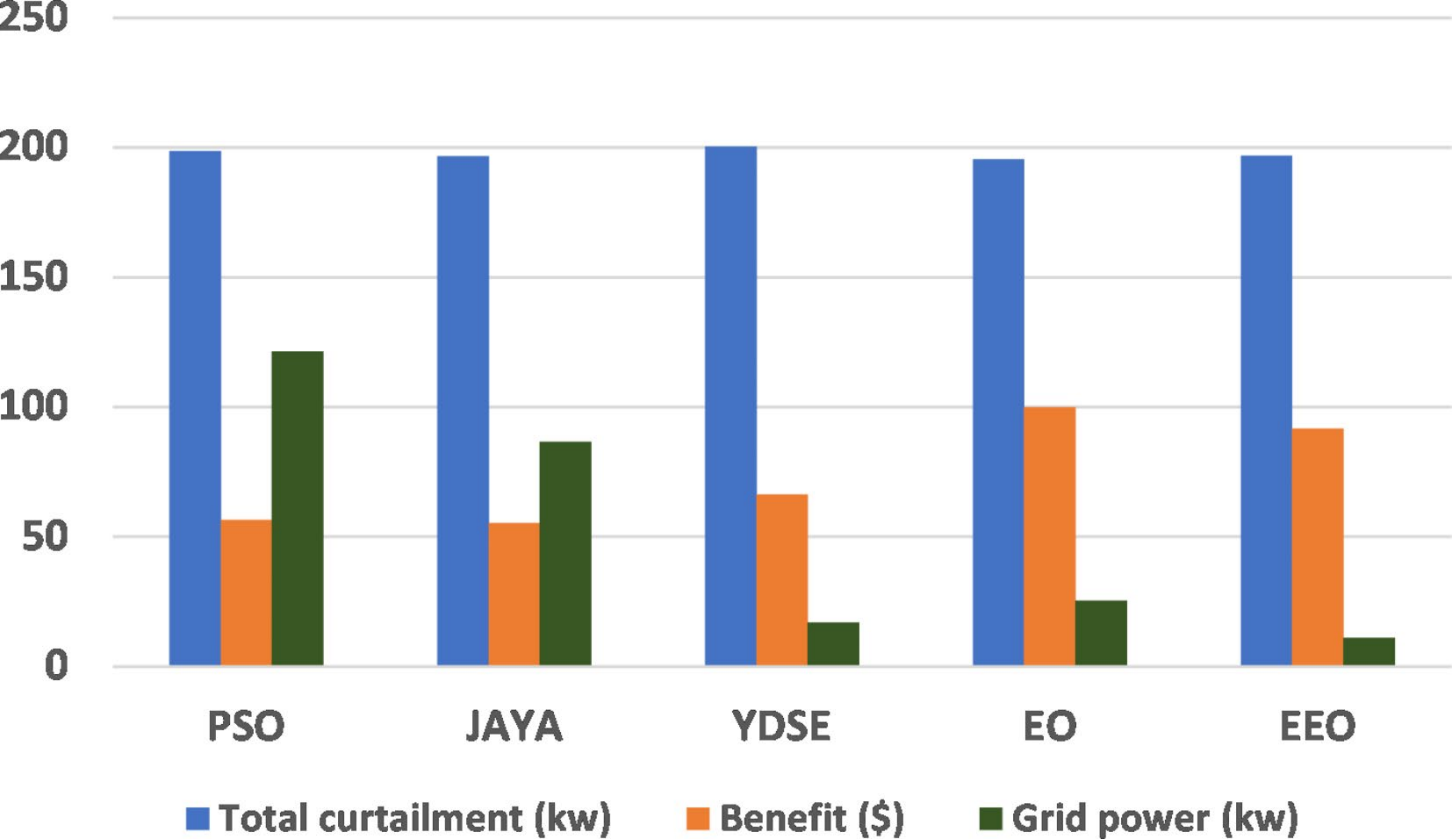
All studied techniques comparison.

To assist the performance of the EEO in solving the MOO problem, the results obtained are compared with those obtained by well-known as PSO^57^, JAYA^58^, recently developed as Young’s double-slit experiment (YDSE)^59^, and the original EO optimization algorithms. A 20 independent runs are performed and the results are compared in Table 6. It can be noticed that using EEO technique yields the best result in solving the EM in term of worst, best, and mean value for the operating cost. The best results for all techniques are then selected and a comparison for all studied techniques based on the power curtailed from all customers, the CO benefit from applying the DRP, and the net power transacted with the utility grid is shown in Fig. 5. It can be noticed the EEO achieves good results in comparison with other techniques.

**Table 6:** Comparison of operating cost for all techniques.

Technique	Worst	Best	Mean
PSO^57^	1473.23	810.04	1268.42
JAYA^58^	1069.208	843.5845	943.5821
YDSE^59^	920.5803	500.5188	714.7552
EO^53^	814.8562	550.0379	674.865
EEO	749.249	483.8306	609.6359

**Fig. 6:**
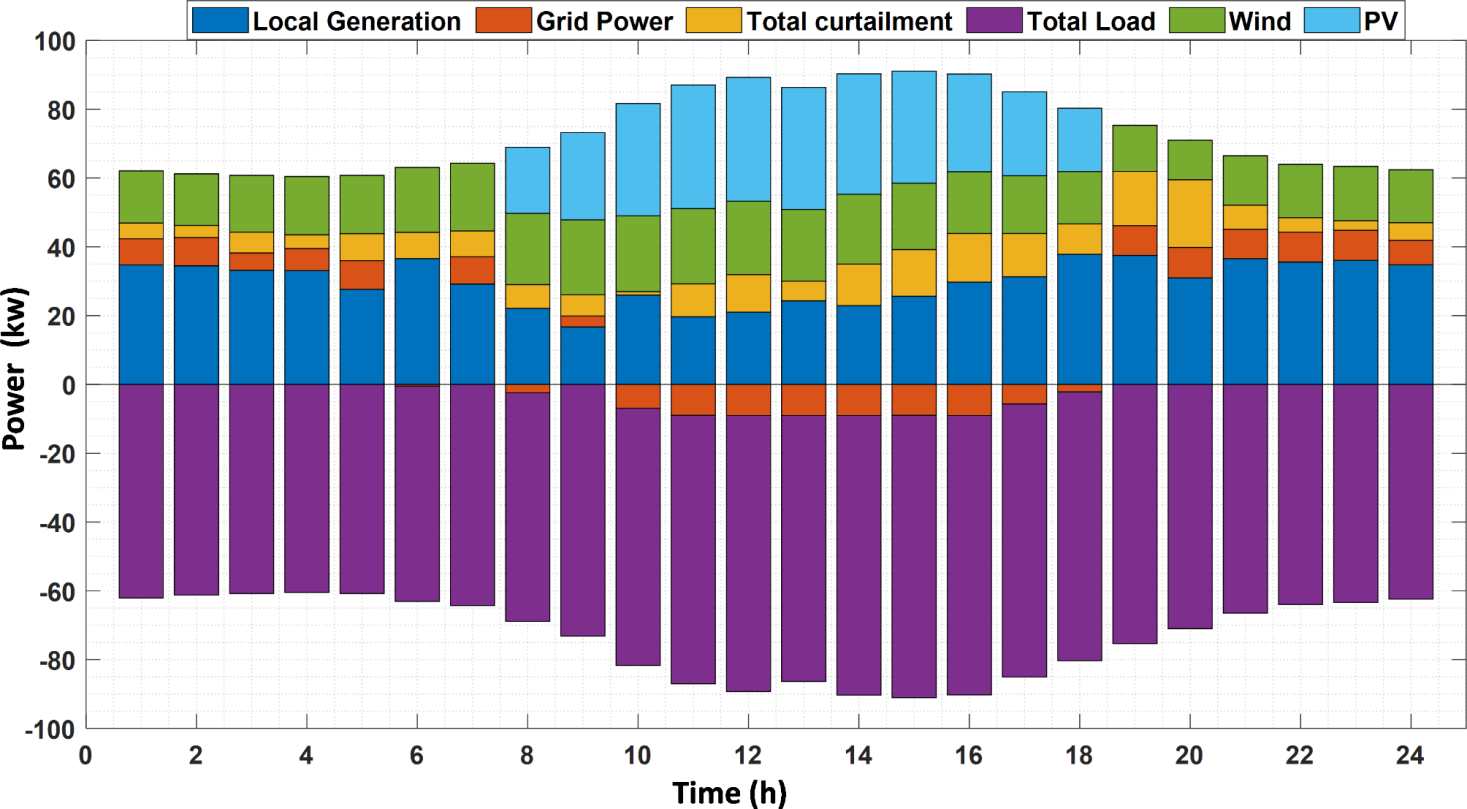
Optimal Day-ahead scheduling of MMG resources.

**Fig. 7:**
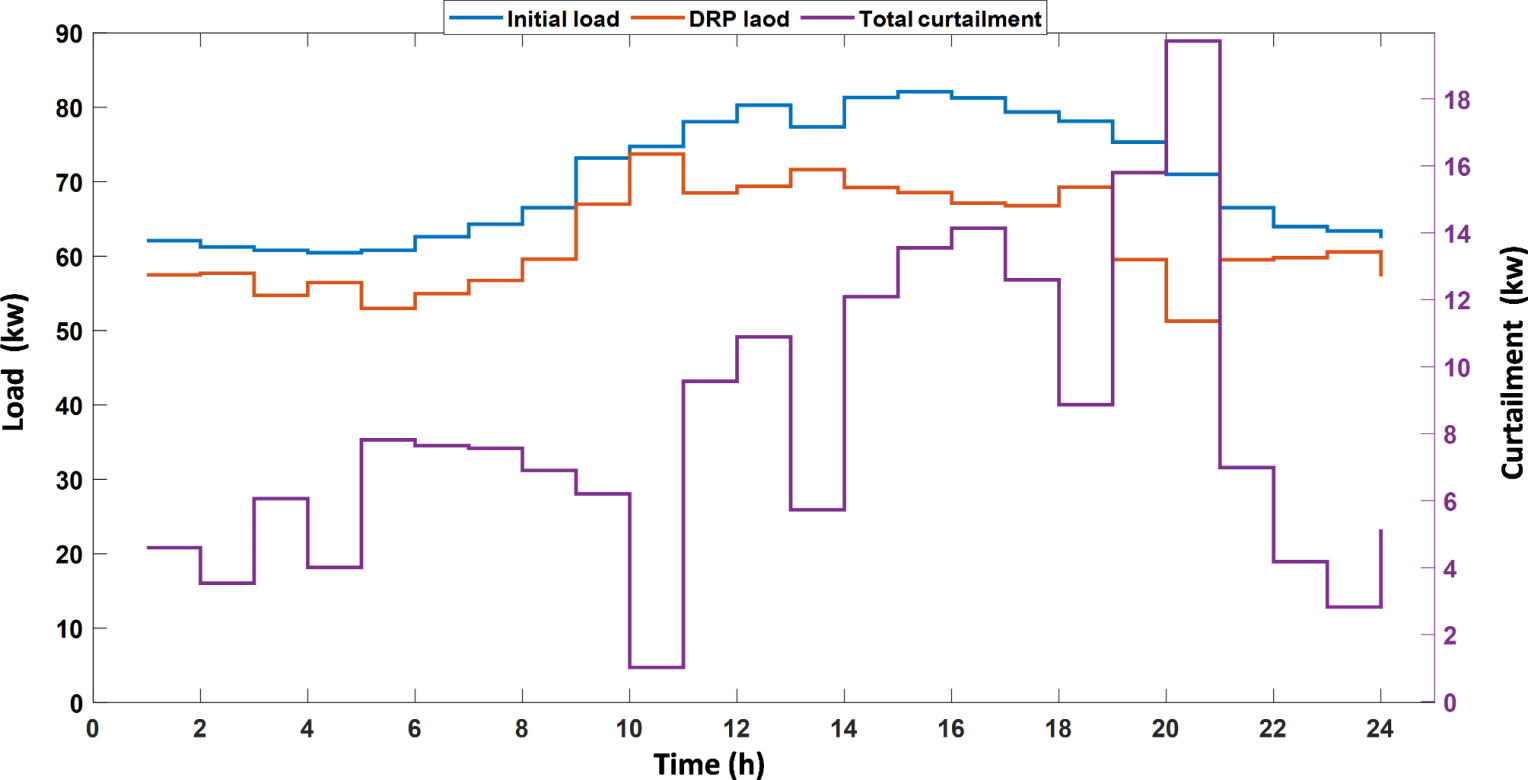
Initial Load demand of the three MG, load with DR and the total curtailment.

Then the first layer (economic layer) is simulated based on the proposed EEO and the original EO techniques; by using the weighted-sum technique to solve the multi-objective problem, the weights are sited as $$\:{w}_{1}={w}_{2}=0.5$$. Using EEO, the day-ahead scheduling for the MMG resources is shown in Fig. 6 ; the negative power direction means that the power is transacted from the MMG to the UG. It can be noticed that at the period of PV and WT generation from hour 11 to hour 18 there is excess power so the power is exported to the UG. The electrical equilibrium between consumption and generation can be noticed for the whole period. The total load from the three MGs before applying the DRP, load with application of DRP, and the total curtailment is shown in Fig. 7. The performance of the first layer is demonstrated in Table 7.. In this layer, the focus is only on the economic objectives. The operating cost acieved by EEO in this Case is $ 556.93 whice is lower than that of EO; also, the MMG benefit in the case of EEO is lower than EO with value of $ 105.21; the other objectives are not optimized, and evaluated based on the optimal scheduling for this first layer. By applying EEO, the total greenhouse gas emissions by EEO are 574.51 kg, and the independence index is 88.6%.

**Table 7:** Performance of the first layer.

	$$\:cost$$ ($)		$$\:{B}_{0}$$ ($)		$$\:\sum\:\left|{P}_{{UG}_{t}}\right|$$		$$\:POL$$ (kg)		$$\:IPI$$ (%)	
	EEO	EO	EEO	EO	EEO	EO	EEO	EO	EEO	EO
First Layer	**556.93**	625.46	**105.21**	100.64	198.66	181.93	574.51	**574.07**	**88.662**	87.75

*Bold indicates the best results.

- **Case II: MLMO framework EM**

For this case study, the economic, environmental, and technical aspects are considered in a sequential manner. The first layer, which consists of two objectives related to economic considerations, is optimized using the approach outlined in Case I. Following this, the environmental and technical layers are optimized. The optimal schedule obtained in the first layer serves as the basis for the second layer’s optimization, which incorporates the optimal environmental objective. The results of Case II based on EEo and EO algorithms is shown in ‎Table 8. In case of EEO, the emissions have been enhanced by -2.45% and − 2.23% over than the first layer for EEO and EO, respectively; however, trying to reduce the emissions results in a slight increase in the operating cost. This is because of the reduction of the imported power from the grid, which initiate the need to generate more power from local conventional DGs with higher generation costs as the ones with lower generation costs were already used. The operating cost in the case of EEO is increased by 3%, which is lower than that achieved by EO by 1.98%.

The third layer uses the scheduling for the second layer and alerts it to consider the technical objectives of this layer ($\:IPI$ maximization); the optimal scheduling is then calculated and demonstrated in ‎Table 8.

**Fig. 8:**
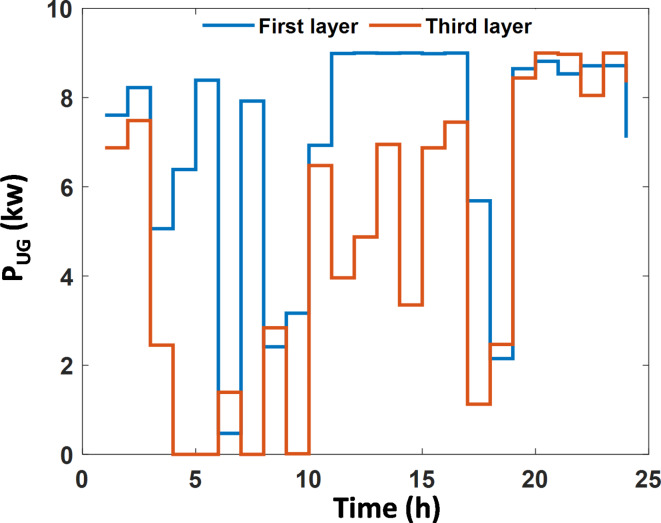
The hourly transacted power with the utility grid.

By EEO, the operating cost is kept the same as the second layer and slightly increased in case of EO; however, the benefit is reduced by 1.24%. Solving the problem of maximizing the independence by EEO through reducing the absolute power transaction with the grid results in extra enhancement in the greenhouse gasses emissions by + 2.4%, this because the power from the grid has higher pollution coefficients. The absolute power transaction with the UG by applying EEO is shown in ‎Figure 8 ; It can be noticed that the power transaction after third layer optimization is lower than that in the first layer. By EEO the $\:IPI$ in this layer is enhanced by + 2.94%, while a higher enhancement in IPI is achieved by EO with + 3.09%.

Also a comparison of the performance of the first case study (one layer) with the second case study (three layers) is shown in ‎Table 8. The operating cost increased slightly from the first case study with about 3% and 4.99% increase for EEO and EO, and the benefit decreased by -4.99% for both algorithms. This to get the optimal condition that minimizes the emissions and maximizes the independence. $\:POL$ has reduced by 4.8% by EEO and only 2.26% by EO. However, the enhancement in the $\:IPI$ achived by EO is higher than EEO by 0.12%, the total IPI is the highest in applying the EEO algorithm.

**Table 8:** Performance of the proposed MLMO technique.

	Second Layer				Third Layer				Total Improvement(%)	
	Result		Improvement(%)		Result		Improvement			
	EEO	EO	EEO	EO	EEO	EO	EEO	EO	EEO	EO
$$\:cost$$($)	573.64	656.67	+ 3	+ 4.98	573.64	656.7	-	+ 0.0045	+ 3	+ 4.99
$$\:B$$($)	101.21	95.95	-3.8%	-4.23	99.954	95.61	-1.24	-0.35	-4.99	-4.99
$$\:\sum\:\left|{P}_{{UG}_{t}}\right|$$(kW)	159.18	173.08	-	-	116.37	129.65	-	-	-	-
$$\:POL$$(kg)	560.40	561.65	**-2.45**	-2.23	547.00	561.12	+ 2.4	+ 0.094	-4.8	-2.26
$$\:IPI$$(%)	89.223	88.28	+ 0.63	+ 0.53	92.165	91.37	+ 2.94	**+ 3.09**	+ 3.5	+ 3.62

* (+ )increase, (-) decrease.

- **Case 3: Probabilistic EM for MMG**

**Fig. 9:**
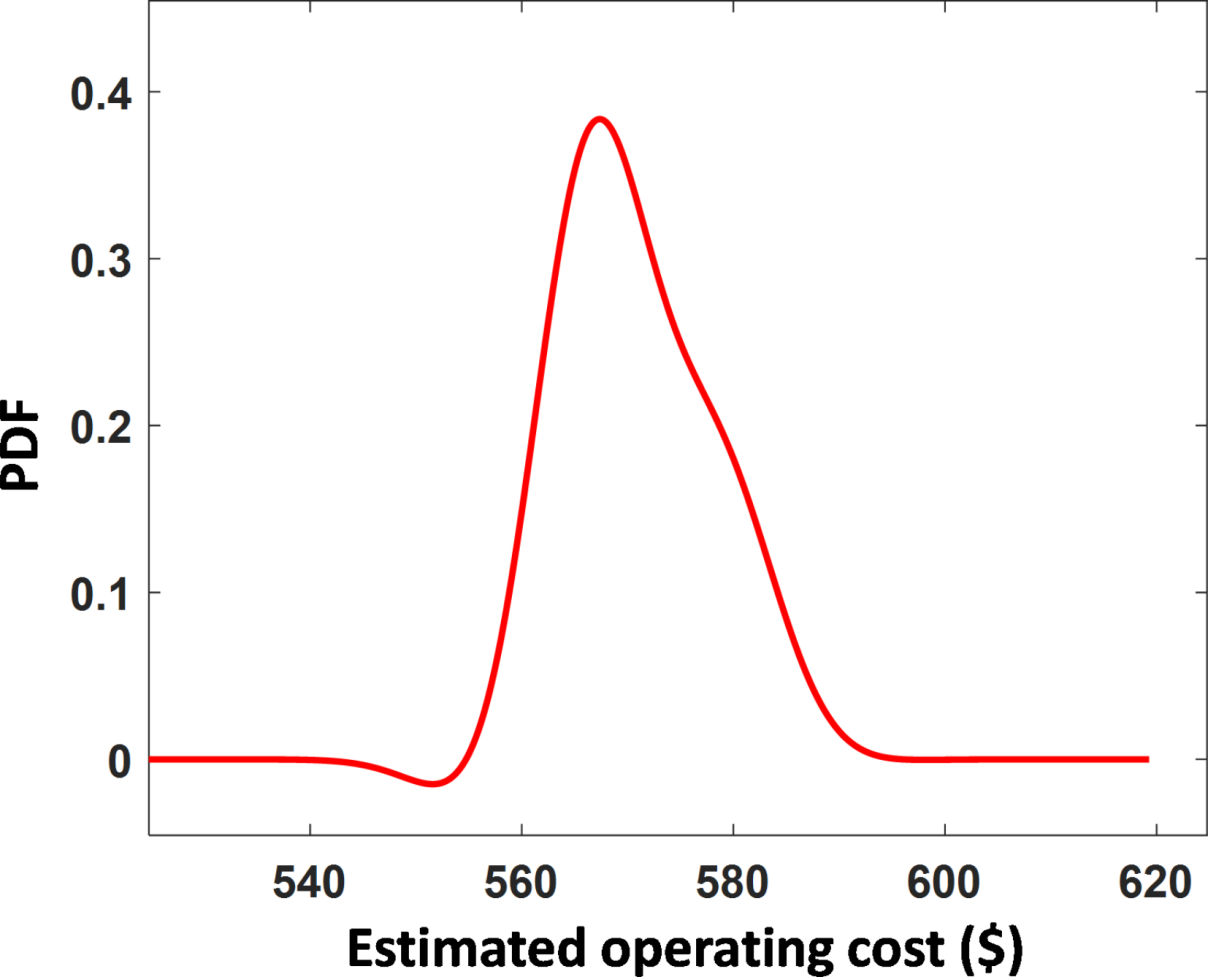
The probability density function of MMG operating cost.

In this scenario the uncertainties of random input variables are considering in solving the EM problem. A combination of 2 m + 1 PEM and EEO algorithm is employed in the EMS for obtaining the optimal solution. In this paper the uncertainties of PV, WT, load demand, and energy cost are considered as uncorrelated IRV and the operating cost is considered as the random output variable. These random variable are represented by the prober PDF as discussed in Section "Generating PDF for Uncertainty variables", WT power, PV power, and market prices are assumed to have a SD of 5% while the load demand is assumed to have SD of 3%^52^. Then the probabilistic EM is solved. The location of the IRVs is tableted in Tables 9 and 10. Then based on these locations EEO is used to solve the probabilistic EM problem based on 2 m + 1 as a separated deterministic problem as it is discussed in section ‎"Uncertainty Modeling". The results obtained from the 2 m + 1 PEM for the moments’ vector are depicted in Table 11. Using Gram–Charlier series the PDF of the operating cost for the MMG is plotted in Fig. 9. The mean cost in case of probabilistic determination is about 2.6% greater than the deterministic case.

**Table 9:** Locations of load demand and renewable sources for MMG system.

**t** **(h)**	Load						Renewable			
	$\:{\varvec{p}}_{1}$			$\:{\varvec{p}}_{2}$			$\:{\varvec{p}}_{1}$	$\:{\varvec{p}}_{2}$	$\:{\varvec{p}}_{1}$	$\:{\varvec{p}}_{2}$
	$\:{\:\varvec{p}}_{\varvec{S}\varvec{M}\varvec{G}\text{1,1}}$ **(KW)**	$\:{\:\varvec{p}}_{\varvec{S}\varvec{M}\varvec{G}\text{2,1}}$ **(KW)**	$\:{\:\varvec{p}}_{\varvec{S}\varvec{M}\varvec{G}\text{3,1}}$ **(KW)**	$\:{\:\varvec{p}}_{\varvec{S}\varvec{M}\varvec{G}\text{1,2}}$ **(KW)**	$\:{\:\varvec{p}}_{\varvec{S}\varvec{M}\varvec{G}\text{2,2}}$ **(KW)**	$\:{\:\varvec{p}}_{\varvec{S}\varvec{M}\varvec{G}\text{3,2}}$ **(KW)**	$\:{\:\varvec{p}}_{\varvec{W}\varvec{T},1}$ **(KW)**	$\:{\:\varvec{p}}_{\varvec{W}\varvec{T},2}$ **(KW)**	$\:{\:\varvec{p}}_{\varvec{P}\varvec{V},1}$ **(KW)**	$\:{\:\varvec{p}}_{\varvec{P}\varvec{V},2}$ **(KW)**
1	33.48	11.72	20.09	30.18	10.56	18.11	8.31	6.99	0.00	0.00
2	33.03	11.56	19.82	29.77	10.42	17.86	8.24	6.93	0.00	0.00
3	32.79	11.48	19.67	29.55	10.34	17.73	9.07	7.63	0.00	0.00
4	32.61	11.41	19.57	29.39	10.29	17.63	9.32	7.84	0.00	0.00
5	32.79	11.48	19.67	29.55	10.34	17.73	9.32	7.84	0.00	0.00
6	33.77	11.82	20.26	30.43	10.65	18.26	10.35	8.71	0.00	0.00
7	34.68	12.14	20.81	31.26	10.94	18.75	10.79	9.08	0.00	0.00
8	35.87	12.56	21.52	32.33	11.31	19.40	11.38	9.57	8.68	7.30
9	39.48	13.82	23.69	35.58	12.45	21.35	11.96	10.06	11.47	9.65
10	40.32	14.11	24.19	36.34	12.72	21.80	12.10	10.18	14.79	12.43
11	42.11	14.74	25.27	37.95	13.28	22.77	12.02	10.12	16.27	13.67
12	43.31	15.16	25.99	39.03	13.66	23.42	11.74	9.87	16.30	13.70
13	41.73	14.61	25.04	37.61	13.16	22.57	11.45	9.63	16.06	13.50
14	43.87	15.35	26.32	39.53	13.84	23.72	11.16	9.38	15.85	13.33
15	44.29	15.50	26.57	39.91	13.97	23.95	10.63	8.94	14.73	12.39
16	43.84	15.34	26.30	39.50	13.83	23.70	9.87	8.30	12.85	10.81
17	42.81	14.99	25.69	38.59	13.50	23.15	9.20	7.74	11.05	9.29
18	42.15	14.75	25.29	37.99	13.30	22.79	8.36	7.04	8.32	7.00
19	40.64	14.22	24.38	36.62	12.82	21.97	7.36	6.19	0.00	0.00
20	38.29	13.40	22.97	34.51	12.08	20.71	6.29	5.29	0.00	0.00
21	35.87	12.56	21.52	32.33	11.31	19.40	7.92	6.67	0.00	0.00
22	34.50	12.08	20.70	31.10	10.88	18.66	8.52	7.17	0.00	0.00
23	34.19	11.97	20.51	30.81	10.78	18.49	8.66	7.29	0.00	0.00
24	33.66	11.78	20.20	30.34	10.62	18.20	8.45	7.11	0.00	0.00

**Table 10:** Locations of cost of power interruption coefficient $\:{\uplambda\:}$ for all customers.

**t** **(h)**	power interruption coefficient											
	$\:{\varvec{p}}_{2}$						$\:{\varvec{p}}_{2}$					
	$$\:{\:\varvec{p}}_{\varvec{\uplambda\:}1,1}$$ **($)**	$$\:{\:\varvec{p}}_{\varvec{\uplambda\:}2,2}$$ **($)**	$$\:{\:\varvec{p}}_{\varvec{\uplambda\:}3,1}$$ **($)**	$$\:{\:\varvec{p}}_{\varvec{\uplambda\:}4,1}$$ **($)**	$$\:{\:\varvec{p}}_{\varvec{\uplambda\:}5,1}$$ **($)**	$$\:{\:\varvec{p}}_{\varvec{\uplambda\:}6,1}$$ **($)**	$$\:{\:\varvec{p}}_{\varvec{\uplambda\:}1,2}$$ **($)**	$$\:{\:\varvec{p}}_{\varvec{\uplambda\:}2,2}$$ **($)**	$$\:{\:\varvec{p}}_{\varvec{\uplambda\:}3,3}$$ **($)**	$$\:{\:\varvec{p}}_{\varvec{\uplambda\:}4,2}$$ **($)**	$$\:{\:\varvec{p}}_{\varvec{\uplambda\:}5,2}$$ **($)**	$$\:{\:\varvec{p}}_{\varvec{\uplambda\:}6,2}$$ **($)**
1	1.71	4.02	2.93	2.86	3.48	2.32	1.43	3.38	2.47	2.41	2.92	1.95
2	1.52	2.93	2.06	2.23	2.50	1.79	1.28	2.47	1.74	1.87	2.10	1.51
3	2.39	3.48	1.96	2.93	2.72	2.17	2.01	2.92	1.64	2.47	2.28	1.83
4	4.09	2.83	2.06	3.46	2.44	3.08	3.43	2.37	1.74	2.90	2.06	2.58
5	4.89	4.13	2.50	4.51	3.31	3.69	4.11	3.47	2.10	3.79	2.79	3.11
6	5.11	1.85	0.76	3.48	1.30	2.93	4.29	1.55	0.64	2.92	1.10	2.47
7	5.48	2.50	1.52	3.99	2.01	3.50	4.60	2.10	1.28	3.35	1.69	2.94
8	5.81	1.63	0.54	3.72	1.09	3.18	4.89	1.37	0.46	3.13	0.91	2.67
9	7.28	4.67	3.15	5.98	3.91	5.22	6.12	3.93	2.65	5.02	3.29	4.38
10	6.69	5.00	1.74	5.85	3.37	4.22	5.63	4.20	1.46	4.91	2.83	3.54
11	6.93	3.80	4.67	5.37	4.24	5.80	5.83	3.20	3.93	4.51	3.56	4.88
12	7.41	4.56	5.22	5.99	4.89	6.31	6.23	3.84	4.38	5.03	4.11	5.31
13	7.93	4.67	5.54	6.30	5.11	6.74	6.67	3.93	4.66	5.30	4.29	5.66
14	8.48	6.85	5.87	7.66	6.36	7.17	7.12	5.75	4.93	6.44	5.34	6.03
15	9.24	3.80	5.98	6.52	4.89	7.61	7.76	3.20	5.02	5.48	4.11	6.39
16	7.71	5.76	6.63	6.74	6.19	7.17	6.49	4.84	5.57	5.66	5.21	6.03
17	7.39	5.76	6.08	6.57	5.92	6.74	6.21	4.84	5.12	5.53	4.98	5.66
18	6.85	6.63	6.85	6.74	6.74	6.85	5.75	5.57	5.75	5.66	5.66	5.75
19	6.30	2.83	4.89	4.56	3.86	5.60	5.30	2.37	4.11	3.84	3.24	4.70
20	4.56	3.91	4.56	4.24	4.24	4.56	3.84	3.29	3.84	3.56	3.56	3.84
21	4.13	4.56	4.24	4.35	4.40	4.18	3.47	3.84	3.56	3.65	3.70	3.52
22	3.27	4.13	3.48	3.70	3.80	3.37	2.75	3.47	2.92	3.11	3.20	2.84
23	2.75	2.50	3.04	2.62	2.77	2.90	2.31	2.10	2.56	2.21	2.33	2.43
24	1.54	4.13	4.56	2.84	4.35	3.05	1.30	3.47	3.84	2.38	3.65	2.57

**Table 11:** Statistical moments of expected cost for case 3.

$$\:E\left(Z\right)$$ ($)	$$\:E\left({Z}^{2}\right)$$ ($)^2^	$$\:E\left({Z}^{3}\right)$$ ($)^3^	$$\:E\left({Z}^{4}\right)$$ ($)^4^	$$\:{\mu\:}_{Z}$$($)	$$\:{\sigma\:}_{Z}$$($)
571.2475	3.2863e + 05	1.9046e + 08	1.1122e + 11	378.9548	129.0465

## Conclusion

This paper has proposed a three-layer, four-objective framework for Energy Management in cooperative MMGs based on a hybrid ε-lexicography–weighted-sum technique. The proposed framework eliminates the need to normalize objectives and simultaneously optimizes four different objectives: operating cost, CO benefit, environmental emissions, and independence from the utility grid. The four objectives of the proposed MLMO framework are operating cost minimization, CO benefit maximization, environmental emissions minimization, and independence from the utility grid enhancement. The enhanced equilibrium optimizer algorithm is used to solve the EM problem in the MMG. Simulation results show that the EEO outperforms other well-known techniques such as PSO, JAYA, YDSE, and the original EO algorithms. Furthermore, the uncertainties of solar power, wind power generation, load demand and the energy prices are considered and the probabilistic EM problem is solved based on 2 m + 1 PEM.Three case studies are developed for the cooperative MMG. In Case I, the first layer is optimized to minimize the operating cost and maximize the benefit, without violating the operating constraints. In Case II, the second and third layers are optimized to reduce greenhouse gas emissions while considering the operating cost and benefit limits determined in the first layer. The third layer enhances the MMG’s independence from the grid. The results show that the proposed framework can achieve significant reductions in greenhouse gas emissions (2.45% and 3.5%), and improvements in independence index (2.49% and 4.8%) in the third layer alone and as a total enhancement, respectively. Overall, the proposed framework provides an effective and efficient solution for multi-objective EM problems in MMGs. In Case III, the 2 m + 1 PEM is used to solve the EM in a probabilistic formulation. The PDF of the operating cost is estimated to give the decision maker a view of the effect of the uncertain variable on the MMG system states. The mean value of the probabilistic framework was increased by about 2.6% over the deterministic framework.

## Data Availability

All data generated or analysed during this study are included in this published article.

## Indices and sets

*j, J *: Index and the total number of consumers
*i, I*: Index and the total number of Diesel generator generators
t, T: Index and number of time intervals in the scheduling horizon

## Parameters and variables

${\gamma}_{t}$: Locational Marginal Prices
${\text{A}}_{\text{c}}$: PV array area(${m}^{2}$)
$${\text{I}}_{{\text{P}\text{v}}_{\text{t}}}$$: Irradiation ($$w/{m}^{2}$$)
$${{\upeta}}_{\text{p}\text{v}}$$: PV’s Efficiency
$$v$$: Wind speed ($$m/s$$)
${P}_{nom}$: Nominal wind power
${v}_{cut-in}$: Cut-in wind speed
${v}_{cut-out}$: Cut-out wind speed
${v}_{nom}$: Nominal wind speed
$\:{\text{a}}_{\text{i}},\:{\text{b}}_{\text{i}}$: Generation cost coefficients of generator$\:i$
$\:{\theta\:}_{j}$: Customer$\:j$ type between 0 and 1
$$\:{c}_{\begin{array}{c}{1}_{j}\\\:\:\end{array}},{c}_{\begin{array}{c}{2}_{j}\\\:\:\end{array}}$$: Customer$\:j$ cost coefficients
$\:{\gamma\:}_{{i}_{k}}$: Pollutant$\:k$ emission coefficient for diesel generator$\:i$
$\:{{\upgamma\:}}_{{\text{U}\text{G}}_{\text{k}}}$: Pollutant$\:k$ emission coefficient of utility grid
$\:\delta\:,\:\sigma\:,\:and\:\beta\:$: Objectives limiting factors
$\:{L}_{t}$: Hourly Total demand
$\:{P}_{{i}_{min}} and \:{P}_{{i}_{max}}$: Minimum and maximum generation limits of generator$\:i$
$\:{RU}_{i}\: and \:{RD}_{i}$: Ramp up and down rates of generator$\:i$
$\:{P}_{{UG}_{max}}$: Maximum rate of power transaction with the utility grid
$\:{CL}_{j}$: Daily curtailment limit of customer$\:j$
$\:UBL$: Daily budget limit

## Variables

$\:{\text{P}}_{{\text{U}\text{G}}_{\text{t}}}$: Utility grid power
$\:cos{t}_{t}^{UG}$: Grid power cost
$\:{P}_{W{T}_{t}}$: Hourly wind turbine power
$\:{{\text{P}}_{\text{P}\text{V}}}_{\text{t}}$: Hourly PV power
$\:{P}_{{i}_{t}}$: Diesel generator$\:i$ power at time$\:t$
$\:cos{t}_{i}^{DG}$: Generation cost of Diesel generator$\:i$
$\:Cos{{t}_{j}}^{cust}$: Customer$\:j$ cost function
$\:{{P}_{c}}_{j}$: Customer$\:j$ power curtailment
$$\:{B}_{1,j}$$: Customer$$\:j$$ benefit
$$\:{\text{B}}_{0}$$: MMG benefit
$\:{f}_{1},{f}_{2,},{f}_{3}and\:{f}_{4}$: First, second, third, and fourth objective functions
$\:cos{t}^{*}$: Operating cost for first layer
$\:{{B}_{0}}^{*}$: Benefit for first layer
$\:{\text{w}}_{1}\text{a}\text{n}\text{d}\:{w}_{2}$: Are the weighting factors of objective function$\:{f}_{1}$
$$\:Pol$$: Environmental emissions
$\:Po{l}^{*}$: Environmental emissions for second layer
$\:{f}_{{G}_{PV}}$: Probability density function of PV
$\:{f}_{{v}_{WT}}$: Probability density function of WT
$\:{\xi\:}_{L,k}$: Standard locations of$\:L$ variable
$$\:{p}_{L,k}$$: Variable$$\:L$$ locations
$\:{w}_{L,k}$: Wight of standard location for variable$\:L$
$\:{\mu\:}_{z}$: Mean of output variable$\:Z$
$\:{\sigma\:}_{z}$: Standard deviation of variable$\:Z$
$\:E\left({Z}^{j}\right)$: The moments vector

## Abbreviations

DR: Demand Response
DSM: Demand Side Management
EM: Energy Management
EMS: Energy Management System
ENS: Energy not Supplied
IDRP: Incentive Demand Response
IPI: Independency Index
MG: Microgrid
MGO: Microgrid operator
MILP: Mixed integer linear programing
MINLP: Mixed integer non-linear programing
MLMO: Multi-layer Multi-objective
MMG: Multi-Microgrid
PDRP: Price-based Demand Response
PSO: Particle swarm optimization
MCS: Monte Carlo Simulation
PEM: Point Estimation Method
PV: Photovoltaic
RES: Renewable Energy sources
SG: Smart Grid
SMG: Single Microgrid
UG: Utility grid
WT: Wind Turbine
PDF: Probability density function
